# Inhibition of connexin hemichannels alleviates non-alcoholic steatohepatitis in mice

**DOI:** 10.1038/s41598-017-08583-w

**Published:** 2017-08-15

**Authors:** Joost Willebrords, Bruno Cogliati, Isabel Veloso Alves Pereira, Tereza Cristina da Silva, Sara Crespo Yanguas, Michaël Maes, Veronica Mollica Govoni, Andressa Lima, Daniele Aparecida Felisbino, Elke Decrock, Marina Sayuri Nogueira, Inar Alves de Castro, Isabelle Leclercq, Luc Leybaert, Robim Marcelino Rodrigues, Mathieu Vinken

**Affiliations:** 1 Department of In Vitro Toxicology and Dermato-Cosmetology, Faculty of Medicine and Pharmacy, Vrije Universiteit Brussel, Laarbeeklaan 103, 1090 Brussels, Belgium; 2 Department of Pathology, School of Veterinary Medicine and Animal Science, University of São Paulo, Av. Prof. Dr. Orlando Marques de Paiva 87, 05508-270 São Paulo, Brazil; 3 Department of Basic Medical Sciences, Physiology Group, Ghent University, De Pintelaan 185, 9000 Ghent, Belgium; 4 Department of Food and Experimental Nutrition, Faculty of Pharmaceutical Sciences, University of São Paulo, Av. Prof. Lineu Prestes 580, 05508-270 São Paulo, Brazil; 5 Laboratory of hepatogastroenterology, Institut de Recherche Expérimentale et clinique, Université catholique de Louvain, Avenue Mounier 53, 1200 Brussels, Belgium

## Abstract

While gap junctions mediate intercellular communication and support liver homeostasis, connexin hemichannels are preferentially opened by pathological stimuli, including inflammation and oxidative stress. The latter are essential features of non-alcoholic steatohepatitis. In this study, it was investigated whether connexin32 and connexin43 hemichannels play a role in non-alcoholic steatohepatitis. Mice were fed a choline-deficient high-fat diet or normal diet for 8 weeks. Thereafter, TAT-Gap24 or TAT-Gap19, specific inhibitors of hemichannels composed of connexin32 and connexin43, respectively, were administered for 2 weeks. Subsequently, histopathological examination was carried out and various indicators of inflammation, liver damage and oxidative stress were tested. In addition, whole transcriptome microarray analysis of liver tissue was performed. Channel specificity of TAT-Gap24 and TAT-Gap19 was examined *in vitro* by fluorescence recovery after photobleaching analysis and measurement of extracellular release of adenosine triphosphate. TAT-Gap24 and TAT-Gap19 were shown to be hemichannel-specific in cultured primary hepatocytes. Diet-fed animals treated with TAT-Gap24 or TAT-Gap19 displayed decreased amounts of liver lipids and inflammatory markers, and augmented levels of superoxide dismutase, which was supported by the microarray results. These findings show the involvement of connexin32 and connexin43 hemichannels in non-alcoholic steatohepatitis and, simultaneously, suggest a role as potential drug targets in non-alcoholic steatohepatitis.

## Introduction

Non-alcoholic fatty liver disease (NAFLD) is the most common chronic liver disease, with an estimated prevalence of 25% worldwide^1^. NAFLD represents a spectrum of diseases, ranging from hepatic steatosis to non-alcoholic steatohepatitis (NASH), liver fibrosis, liver cirrhosis and eventually hepatocellular carcinoma^2^. Liver steatosis can be caused by an increased influx of fatty acids through high-fat diet, insulin resistance, drugs and genetic factors. As such, steatosis is featured by triglyceride-based lipid droplet accumulation in the cytosol of hepatocytes^3^. Hepatic steatosis may evolve to NASH in response to a number of triggers, such as inflammatory cytokines, adipokines, reactive oxygen species and endoplasmatic reticulum stress^4^.

NASH is driven by a plethora of intracellular signaling cascades, all which underlie the deposition of fat in hepatocytes and the induction of an inflammatory reaction^5^. Knowledge regarding the involvement of intercellular communication in this process is, however, scarce. Direct intercellular communication is predominantly mediated by gap junctions, which allow the transfer of small hydrophilic molecules, such as adenosine triphosphate (ATP), as well as ions between neighbouring cells. Gap junctions arise from the interaction between 2 hemichannels of adjacent cells, which in turn consist of 6 connexin (Cx) proteins. At present, 21 different Cx species have been identified, all which are expressed in a cell-specific way. In liver, hepatocytes mainly produce Cx32, while the non-parenchymal cell population typically harbours Cx43^6^. However, upon dedifferentiation, as seen in several pathological conditions *in vivo* ^7^ as well as in cultures of primary hepatocytes^8^, a shift from Cx32 to Cx43 expression takes place. In recent years, compelling evidence has shown that Cx hemichannels are not merely structural precursors of gap junctions. Indeed, Cx hemichannels can also establish a pathway for communication on their own, albeit between the cytosol of an individual cell and its extracellular environment. In contrast to gap junctions, Cx hemichannels have a low open probability. They seem to become preferentially activated by pathological stimuli, such as decreased extracellular calcium concentration^9^, mechanical stimulation^10^, oxidative stress^11^, ischemia/reperfusion insults^12^ and inflammatory conditions^13, 14^. We previously reported that Cx32 has a protective role in NASH, as evidenced by higher levels of alanine aminotransferase (ALT), aspartate aminotransferase (AST), inflammatory cytokines and oxidative stress in Cx32^−/−^ mice^15^. However, because of genetic ablation of their common building block Cx32, this approach does not allow to distinguish between communication mediated by gap junctions and hemichannels. For this reason, the present study was set up, in which NASH mice were treated with Gap24 or Gap19 coupled to a transactivator of transcription (TAT) sequence, being peptides that act as specific inhibitors of hemichannels consisting of Cx32 and Cx43, respectively, while leaving their full gap junction channel counterparts unaffected^12^. As the targets of both peptides are located within the cell, they were indeed linked to a HIV-1 TAT-sequence in order to improve cytosolic uptake^16^. To our knowledge, this is the first study exploring the involvement of Cx hemichannels in NASH.

## Results

### Effects of TAT-Gap24 and TAT-Gap19 on gap junction activity and connexin hemichannel responses

**Figure 1:**
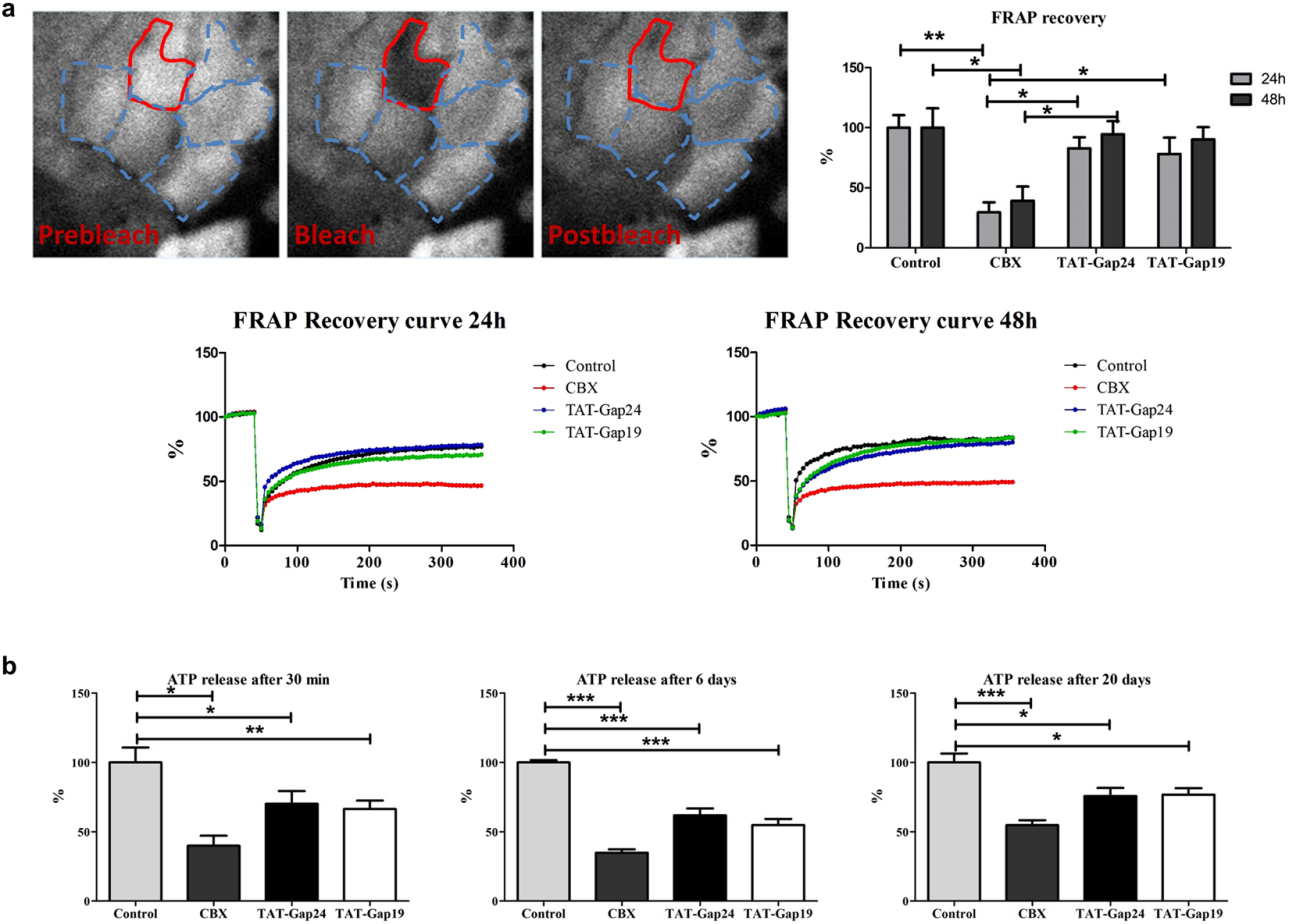
Effects of TAT-Gap24 and TAT-Gap19 on gap junction activity and connexin hemichannel responses. Primary rat hepatocytes were exposed to 50 µM CBX, 20 µM TAT-Gap24, 20 µM TAT-Gap19 or vehicle control. (**a**) Gap junction activity measured through FRAP analysis after 24 h and 48 h (n = 4, N = 4). Fluorescence in the bleached cell was expressed as the percentage of recovery relative to the starting level just before photobleaching. A similar analysis was performed on cells distant from the bleached area to monitor photobleaching outside the targeted region. The recovery values for each experiment were normalized to the corresponding vehicle control condition. Pre-bleach (left panel), bleach (middle panel), post-bleach (right panel) after 6 min. (**b**) ATP release 30 min after incubation of TAT-Gap24, TAT-Gap19 or vehicle control for 0 min, 6 days and 20 days (n = 3, N = 6). Data are expressed as means ± SEM with *p < 0.5, **p < 0.01 and ***p < 0.001.

TAT-Gap24 and TAT-Gap19 mimic an amino acid sequence in the intracellular loop region of Cx32 and Cx43, respectively. Gap19 was found to bind to the *C*-terminal area of Cx43, which abolishes the interaction between the intracellular loop and the *C*-terminal area, in turn inhibiting Cx43 hemichannel opening^12^. A similar mechanism is believed to underlie the actions of TAT-Gap24 on Cx32 hemichannels. In order to assess the specificity of TAT-Gap24 and TAT-Gap19 to block Cx hemichannels, but not gap junctions, fluorescence recovery after photobleaching (FRAP) analysis was performed in primary rat hepatocyte cultures (n = 4, N = 4). Specifically, 24 h after plating, rat hepatocytes were exposed to 20 µM TAT-Gap19, 20 µM TAT-Gap24, 50 µM carbenoxolone (CBX), a well-known inhibitor of Cx channels^17^, or vehicle control for 24 h and 48 h. When applied at 20 µM, TAT-Gap24 and TAT-Gap19 did not induce cytotoxicity in primary rat hepatocyte cultures (Supplementary Fig. 1). CBX significantly reduced fluorescence recovery after 24 h (p < 0.01) and 48 h (p < 0.05) compared to vehicle control. This effect was not seen in cell cultures exposed to TAT-Gap24 or TAT-Gap19 (Fig. 1a). Plateau, half-time and mobile fraction values supported these results (Table 1).

**Table 1:** Calculations of FRAP curve parameters following a non-linear curve fit of control, carbenoxolone, TAT-Gap24 and TAT-Gap19 after 24 h and 48 h of exposure.

Parameter	Control		Carbenoxolone		TAT-Gap24		TAT-Gap19	
Exposure time (h)	**24**	**48**	**24**	**48**	**24**	**48**	**24**	**48**
Y_0_ (%)	36.74	54.16	33.23	34.42	48.72	41.82	39.59	41.59
Plateau (%)	76.54	82.36	47.51	48.63	77.30	79.64	69.79	82.54
Tau (s)	67.25	51.53	42.30	46.62	62.63	76.86	61.69	65.60
Half-time (s)	46.62	35.72	29.32	32.32	43.41	53.27	42.76	45.47
Mobile fraction (%)	39.81	28.21	14.28	14.21	28.58	37.82	30.20	40.95

It has been well documented that Cx hemichannels can open upon low extracellular Ca^2+^ concentrations^18^. Accordingly, a divalent-free buffer was used to trigger their opening. In view of indirectly testing peptide stability, TAT-Gap24 and TAT-Gap19 were first incubated at 37 °C for 0 min, 6 days or 20 days in a classic incubator. After each time point, extracellular ATP release, a measure of Cx hemichannel activity, was recorded in cultivated hepatocytes after 30 min of exposure (n = 3, N = 6). It was found that TAT-Gap24 significantly reduces ATP levels after incubation at 37 °C in comparison with vehicle control after 30 min (p < 0.01), 6 days (p < 0.001) and 20 days (p < 0.001). The same holds true for TAT-Gap19, which reduced extracellular ATP levels after 30 min (p < 0.05) incubation of the peptides (Fig. 1b), thus showing sustained functionality of both peptides.

### Effects of TAT-Gap24 and TAT-Gap19 on connexin expression in liver tissue

**Figure 2:**
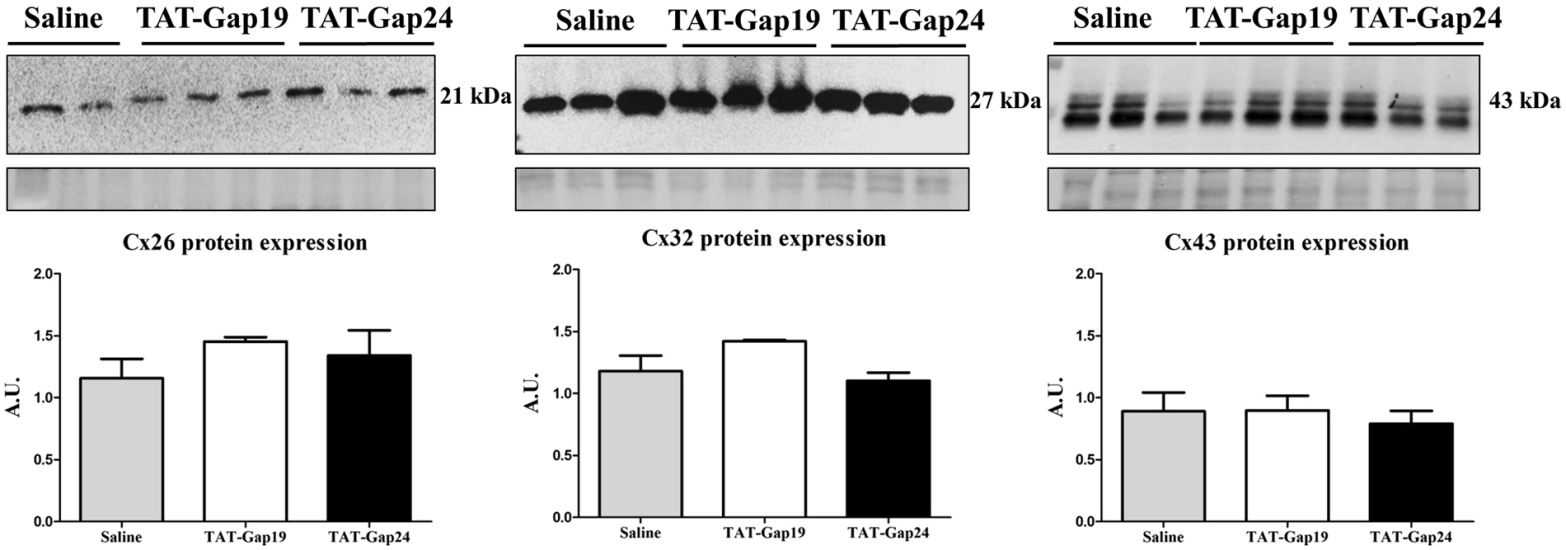
Effects of TAT-Gap24 and TAT-Gap19 on connexin protein expression in liver. After 8 weeks of CHFD, an osmotic pump was surgically implanted in the abdominal cavity, which ensured sustained release of 1 mg/kg/day TAT-Gap24 (n = 11) or TAT-Gap19 (n = 12) or saline (n = 14) for another 2 weeks, while continuing the diet. Immunoblot analysis of Cx26 (21 kDa), Cx32 (27 kDa) and Cx43 (43 kDa) after separation and blotting, after which results were normalized to total protein loading. Blot images are cropped. Full-length blots are presented in Supplementary Fig. 4. Data are expressed as means ± SEM.

In mice fed a choline-deficient high-fat diet (CHFD) (n = 3), Cx32 mRNA expression was decreased (p < 0.01), while Cx32 protein expression was elevated (p < 0.05) in comparison with normal diet (ND)-fed animals (n = 3). It has been shown that Cx32 mRNA degradation occurs through shortening of the poly(A) tail during inflammation^19^, while Cx32 protein expression is often increased upon inflammation in mice and in liver of hepatitis patients^20, 21^. Cx26 and Cx43 expression remained unchanged (Supplementary Figs 2 and 3). In order to verify that administration of the Cx hemichannel inhibitors in mice does not affect protein expression of Cx26, Cx32 and Cx43 in liver, immunoblot analysis of liver extracts was performed. No effects of the peptides were found on Cx26, Cx32 and Cx43 protein expression in TAT-Gap24-treated (n = 3) and TAT-Gap19-treated mice (n = 3) in comparison with saline-treated animals (n = 3) (Fig. 2 and Supplementary Fig. 4).

### Effects of TAT-Gap24 and TAT-Gap19 on biometric parameters, liver histology and serum transaminases

**Figure 3:**
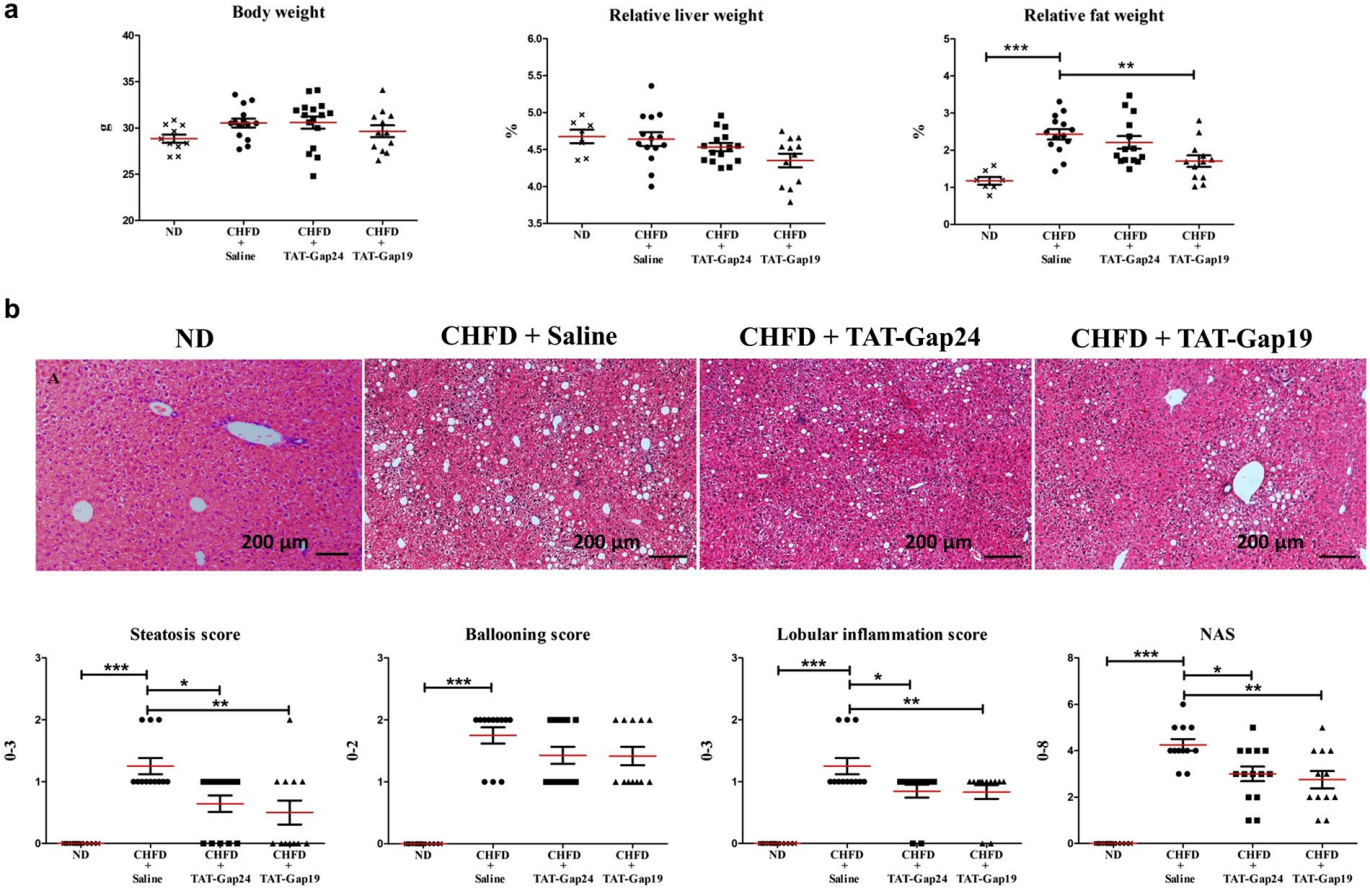
Effects of TAT-Gap24 and TAT-Gap19 on biometric parameters and liver histology in NASH. After 8 weeks of CHFD, an osmotic pump was surgically implanted in the abdominal cavity, which ensured sustained release of 1 mg/kg/day TAT-Gap24 (n = 11) or TAT-Gap19 (n = 12) or saline (n = 14) for another 2 weeks while continuing the diet. (**a**) Body, fat and liver of mice and relative liver and fat weight. (**b**) Steatosis, lobular inflammation, ballooning and NAS score based on hematoxylin-eosin staining of liver tissue of ND group (first panel), saline group (second panel), TAT-Gap24 (third panel) and TAT-Gap19 group (fourth panel). Data are expressed as means ± SEM with *p < 0.5, **p < 0.01 and ***p < 0.001.

**Figure 4:**
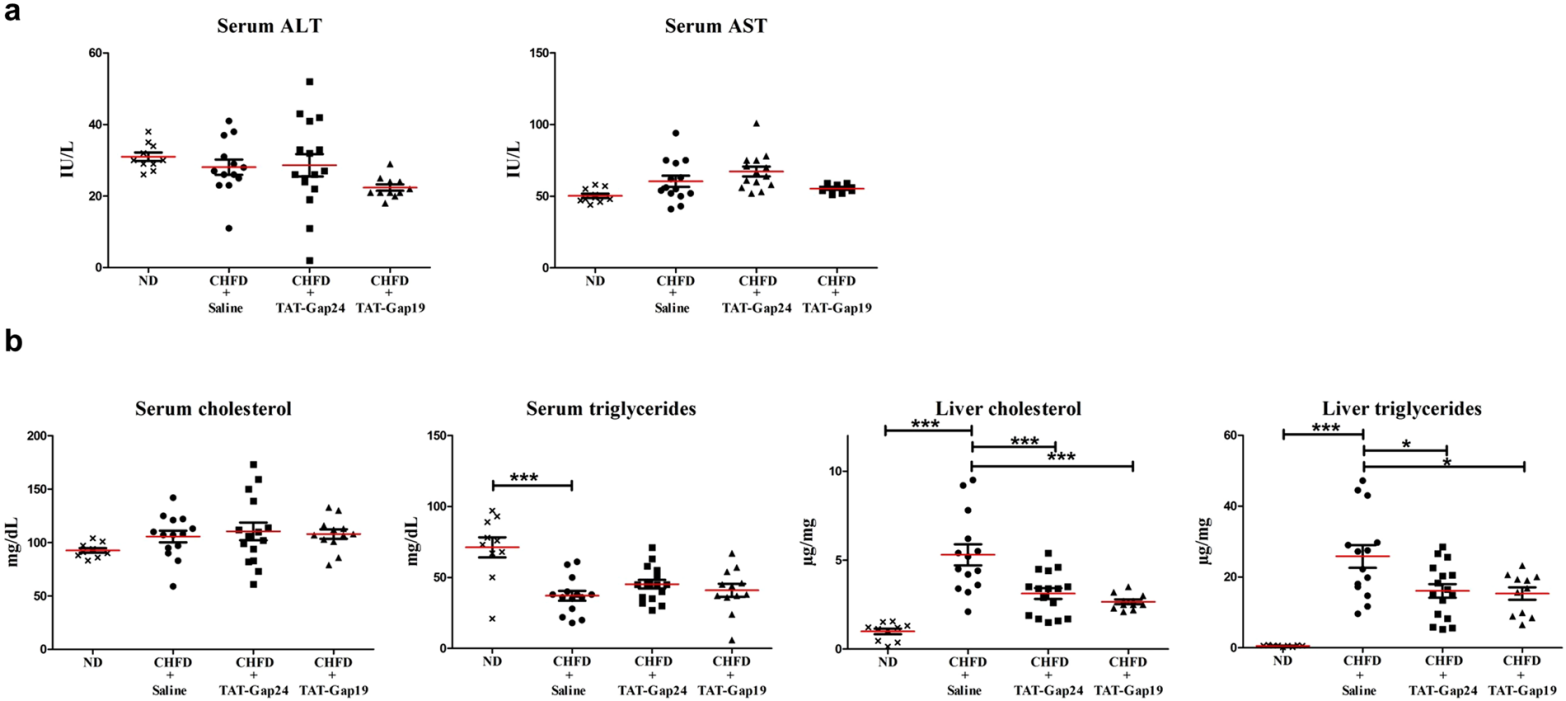
Effects of TAT-Gap24 and TAT-Gap19 on serum transaminases and serum and liver lipid content in NASH. After 8 weeks of CHFD, an osmotic pump was surgically implanted in the abdominal cavity, which ensured sustained release of 1 mg/kg/day TAT-Gap24 (n = 11) or TAT-Gap19 (n = 11) or saline (n = 13) for another 2 weeks while continuing the diet. (**a**) Serum ALT and AST. (**b**) Serum and liver triglycerides and cholesterol. Data are expressed as means ± SEM with *p < 0.05 and ***p < 0.001.

Relative fat weight more than doubled and was significantly higher in CHFD-fed mice (n = 14) in comparison with ND-fed counterparts (p < 0.001) (n = 10). This effect was partly diminished in TAT-Gap19-treated animals (p < 0.01) (n = 12) in comparison with saline-treated mice (n = 14). No differences were seen in TAT-Gap24-treated animals (n = 11) (Fig. 3a). Evaluation of liver biopsy specimens is still considered as the gold standard for diagnosing NASH. In particular, histopathological NASH assessment is typically based on scoring of steatosis, lobular inflammation and hepatocellular ballooning^22^. All scores were 0 in the ND-fed group (n = 10) and there was an augmentation of steatosis score (p < 0.001), ballooning score (p < 0.001), lobular inflammation score (p < 0.001) and NAFLD activity score (NAS) (p < 0.001) in CHFD-fed mice (n = 14) in comparison with the ND-fed group (n = 10). The histopathological steatosis score was significantly reduced in both TAT-Gap24 (n = 14) (p < 0.05) and TAT-Gap19-treated animals (n = 12) (p < 0.01) in comparison with saline mice (n = 12), as clearly seen on hematoxylin-eosin staining (Fig. 3b). In fact, out of 14 mice for TAT-Gap24 and 12 mice for TAT-Gap19, 5 and 7 animals, respectively, showed less than 5% steatosis. Similar results were obtained for lobular inflammation in TAT-Gap24 (n = 14) (p < 0.05) and TAT-Gap19-treated animals (n = 12) (p < 0.01). No significant differences were seen on ballooning. NAS was lower in both treated mouse groups (p < 0.05) (Fig. 3b). No effects of the peptides were found on ALT and AST levels (Fig. 4a).

### Effects of TAT-Gap24 and TAT-Gap19 on serum and liver lipids

A principal hallmark of NASH includes the accumulation of lipid droplets in the cytosol of hepatocytes. These droplets consist of triglycerides and are delineated by a phospholipid monolayer. In particular, liver steatosis is triggered by enhanced *de novo* synthesis of fatty acids or increased influx through diet^3^. Moreover, cholesterol induces hepatocellular sensitivity to inflammatory mediators^23^. In fact, cholesterol auto-oxidizes to diverse oxysterols under inflammatory and upregulated oxidative stress conditions. Oxysterols have been suggested to be involved in NASH pathogenesis^24^. In this context, higher amounts of liver triglycerides (p < 0.001) and cholesterol (p < 0.001) were noticed in CHFD-fed mice (n = 14). However, after administration of TAT-Gap24, lower levels of triglycerides (p < 0.05) and cholesterol (p < 0.01) were found in the liver (n = 11) (Fig. 4b). The same holds true for triglycerides (p < 0.05) and cholesterol (p < 0.001) after TAT-Gap19 administration (n = 11). There were, however, no changes in serum lipid concentrations in treated animals (Fig. 4b).

### Effects of TAT-Gap24 and TAT-Gap19 on cytokines

**Figure 5:**
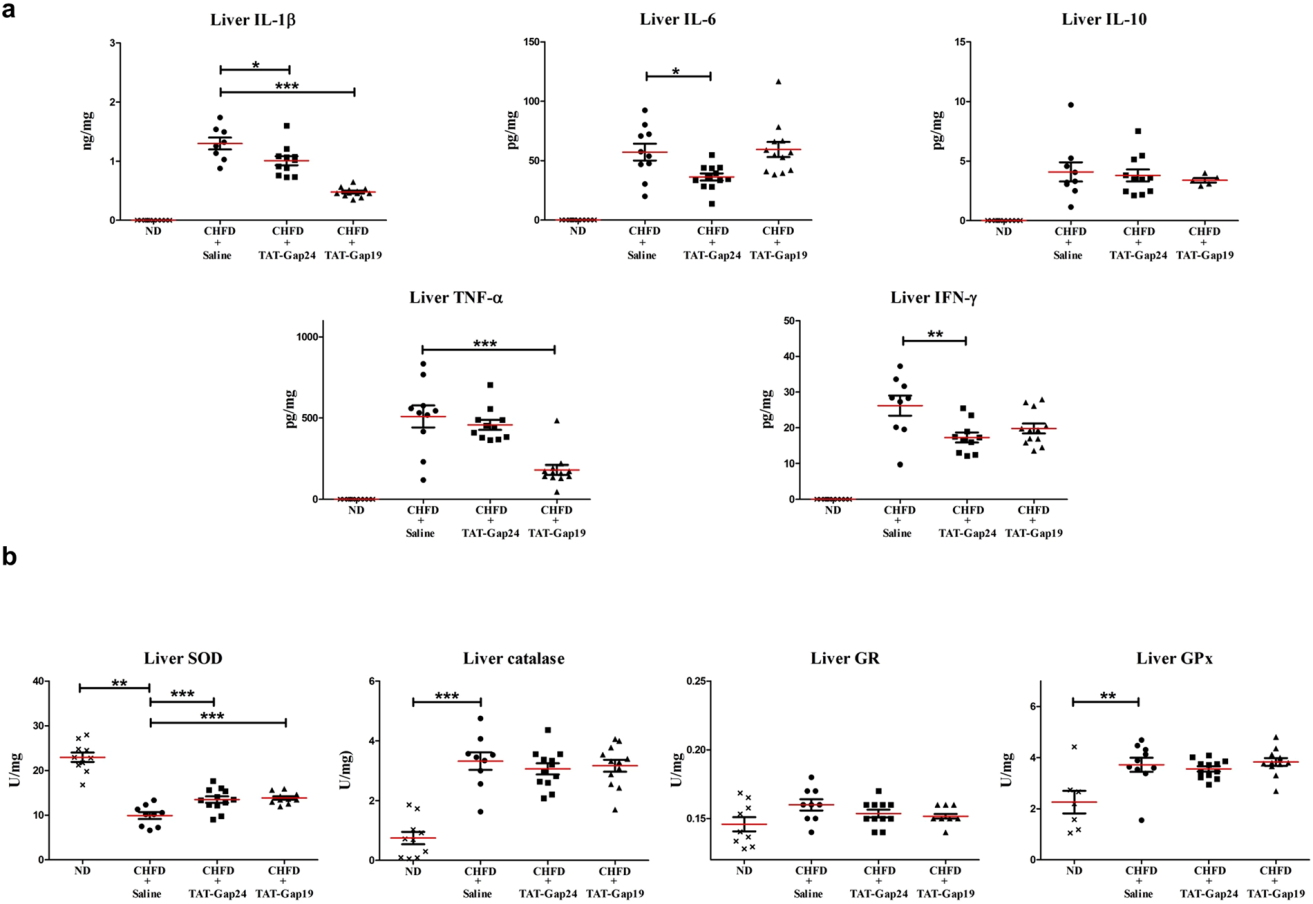
Effects of TAT-Gap24 and TAT-Gap19 on inflammatory cytokines and oxidative stress in NASH. After 8 weeks of CHFD, an osmotic pump was surgically implanted in the abdominal cavity, which ensured sustained release of 1 mg/kg/day TAT-Gap24 (n = 11) or TAT-Gap19 (n = 12) or saline (n = 14) for another 2 weeks while continuing the diet. (**a**) Levels of IFN-γ, IL-6, IL-1β, TNF-α and IL-10 in liver tissue and serum. (**b**) Activity of SOD, GR, GPx and catalase in liver tissue. Data are expressed as means ± SEM with *p < 0.05, **p < 0.01, and ***p < 0.001.

Steatotic hepatocytes can induce cytokine and chemokine production in Kupffer cells with subsequent recruitment and activation of inflammatory cells^25^. Bearing this in mind, a number of pro-inflammatory cytokines, including interleukin (IL)-1β, IL-6, interferon (IFN)-γ and tumor necrosis factor (TNF)-α, and anti-inflammatory cytokines, namely IL-10, which are considered of relevance for controlling hepatic injury-associated inflammation^26^, were monitored in this study. No detectable levels of cytokines could be found in ND-fed mice (n = 10). Statistically significant lower levels of IL-1β (p < 0.05), IL-6 (p < 0.05) and IFN-γ (p < 0.01) were found upon treatment of NASH mice with TAT-Gap24 (n = 11), while levels of TNF-α and IL-10 were unchanged (Fig. 5a). Similar effects were noticed in TAT-Gap19-treated NASH mice (n = 12), which showed decreased amounts of IL-1β (p < 0.001) and TNF-α (p < 0.001).

### Effects of TAT-Gap24 and TAT-Gap19 on oxidative stress

A major contributor to the pathogenesis of NASH is oxidative stress, typified by the excessive production of reactive oxygen species. Superoxide dismutase (SOD), an enzyme responsible for catalyzing superoxide anions, is usually downregulated in the presence of reactive oxygen species, which also holds true in NASH mice in comparison with lean littermates^27^. This equally applies to glutathione reductase (GR), glutathione peroxidase (GPx) and catalase, being anti-oxidant enzymes^28^. In fact, mice and human patients with NASH exhibit decreased glutathione content, SOD and catalase activities^27, 29^. Interestingly, higher levels of GPx (p < 0.01) and catalase (p < 0.001) as well as lower amounts of SOD (p < 0.01) were seen in the liver of mice fed a CHFD (n = 14) in comparison with ND-fed animals (n = 10). The effects of SOD was partially reversed (p < 0.01) in liver of TAT-Gap24-treated (n = 12) and TAT-Gap19-treated mice (n = 12) (p < 0.001) (Fig. 5b).

### Effects of TAT-Gap24 and TAT-Gap19 on the liver transcriptome

**Figure 6:**
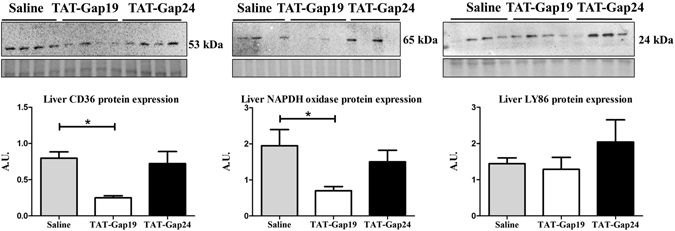
Protein expression analysis of liver tissue of TAT-Gap24 and TAT-Gap19-treated NASH animals. After 8 weeks of CHFD, an osmotic pump was surgically implanted in the abdominal cavity, which ensured sustained release of 1 mg/kg/day TAT-Gap24 (n = 6) or TAT-Gap19 (n = 8) or saline (n = 9) for another 2 weeks while continuing the diet. Immunoblot analysis of CD36 (53 kDa), NADPH oxidase (65 kDa) and LY86 (24 kDa) after separation and blotting, after which results were normalized to total protein. Blot images are cropped. Full-length blots are presented in Supplementary Fig. 5. Data are expressed as means ± SEM with *p < 0.05.

In order to shed more light onto the mechanism by which Cx hemichannel inhibition contributes to a decrease in NASH, whole transcriptome analysis was performed on isolated RNA of liver tissue of TAT-Gap19-treated (n = 8), TAT-Gap24-treated (n = 6), and saline-treated (n = 9) NASH animals, and ND-fed mice. As such, 374 genes were found to be differentially expressed in CHFD-fed mice (n = 9) in comparison with ND-fed littermates. Some of the most important pathways involved include cholesterol biosynthesis (*Hmgcs1*, *Fdps*, *Sqle*, *Nsdhl*) cytochrome P450 oxidation (*Cyp17a1*, *Cyp51*, *Cyp7b1*, *Cyp2f2*), and insulin signaling (*G6pc*, *Aldoc*, *Got1*, *Gys2*), which showed mainly downregulated genes and fatty acid β-oxidation (*Cd36*, *Fabp2*, *Slc27a*), and peroxisome proliferator-activated receptor signaling (*Cd36*, *Lpl*, *Cyp4a14*), which exhibited mostly upregulated genes (Supplementary Table 1). Several genes encoding proteins involved in lipid transport (*Mfsd2a*, *Cd36*), T-cell regulation (*Adgre1*, *Vsig4*, *Clec7a*), general inflammatory response (*Lilr4b*, *Ifi27l2b*, *Cd68*, *Cd5l*, *Ly86*, *Fcer1g*, and *C1qb*), and oxidation (*Hmox1*, *Cybb*, and *Gsta2*) were downregulated, while a gene involved in cholesterol catabolism (*Cyp7b1*) was upregulated in the TAT-Gap19-treated group. Moreover, expression of a number of microRNAs linked to tumour suppression (*Mir15a* and *Mir101b*) was increased (Supplementary Table 2). Differentially expressed genes in the TAT-Gap24 group have more obscure functions (Supplementary Table 3). To substantiate some of the findings at the transcriptional level, a number of gene changes were verified at the translational level using immunoblot analysis. Complying with the microarray data, lower protein levels of cluster of differentiation (CD)36 (p < 0.05), a key protein in hepatic uptake of fatty acids^30^, were detected in liver of TAT-Gap19-treated mice. Similar effects (p < 0.05) were seen at the protein level for nicotinamide adenine dinucleotide phosphate (NADPH) oxidase, a superoxide generating enzyme, encoded by the *Cybb* gene, which forms reactive oxygen species and that plays a role in antigen presentation and the regulation of adaptive immunity^31^. No differences at the protein level were found for lymphocyte antigen (LY)86 (Fig. 6 and Supplementary Fig. 5).

## Discussion

At present, 3–5% of people worldwide are affected by NASH, thus constituting a global health problem^2^. Since NASH is closely associated with obesity, insulin resistance and other metabolic features, the prevalence is anticipated to steeply increase in the upcoming years^32^. Therefore, there is an urgent need for drugs to efficiently counteract NASH. In the last decade, it has become clear that Cx hemichannels can act as pathological pores, as they are opened upon fatty acid exposure^33^, inflammation^14^ and oxidative stress^11^. In the present study, the potential role of Cx32 and Cx43 hemichannels in NASH was investigated. This research relied on the use of TAT-Gap24 and TAT-Gap19, which reproduce specific amino acid modules in the intracellular loop domain of Cx43 and Cx32, respectively. In line with work from other groups using different cell types^12, 18^, TAT-Gap24 and TAT-Gap19 were found to inhibit extracellular ATP release, indicative for Cx hemichannel activation, but not gap junctions, in cultures of primary rat hepatocytes. Concerning FRAP analysis, the recovery plateaus at 48 h are slightly higher than the recovery plateaus at 24 h. In fact, upon 2-step collagenase isolation of hepatocytes from the rat liver, the vast array of intercellular contacts becomes abolished, resulting in the loss of normal cytoarchitecture^34–36^ and gap junctions^37^. After 12 h of cultivation, hepatocytes start to regain their normal morphology, and after about 24 h, *de novo* intercellular contacts reappear^34, 36^. Thereafter, gap junctional intercellular communication is increased at 72 h in comparison with 48 h of cultivation^37^. This effect probably contributes to the higher recovery plateau at 48 h of exposure to carbenoxolone, TAT-Gap24 and TAT-Gap19 in comparison with 24 h. In a next set of experiments, TAT-Gap19 and TAT-Gap24 were tested in a mouse model of NASH. Of note, the *in vitro* results obtained in rat hepatocytes are comparable to the *in vivo* model in mice, as there are only marginal differences between both species in Cx32 and Cx43 amino acid sequences^38, 39^. Importantly, it has been shown in a variety of cell types, such as astrocytes, HeLa cells, T cells and CHO cells^40^, that the TAT sequence can internalize through different receptors, including CD26^41^, lipoprotein receptor-related protein^42^ and CXC chemokine receptor 4^43^. Gap19 has an intracellular target located on the *C*-terminal tail of Cx43 to which it binds with a K_d_ of ~2.5 μM^12^, making it possible that the peptide can be trapped and retained intracellularly. Gap24 is believed to act in a similar way on Cx32^18^. Both TAT-Gap24-treated and TAT-Gap19-treated NASH mice exhibited decreased levels of triglycerides, cholesterol, IL-1β as well as higher quantities of SOD in liver tissue. Moreover, TAT-Gap19 also decreased levels of ALT and TNF-α, while TAT-Gap24-treated mice presented lower IFN-γ and IL-6 levels. It should be stressed, however, that non-specific effects can as such not be fully excluded from this study because of the lack of scrambled peptide control. SOD, catalase, GR and GPx activity levels are usually decreased during NASH^27, 29^, as overproduced reactive oxygen species may directly deplete antioxidant molecules and inhibit the activities of antioxidant enzymes^44^. Since catalase, GPx and SOD perform their enzymatic activity in close relationship to each other^44^, decreases in catalase and GPx activity could reflect a compensatory mechanism to the increased activity levels of SOD.

To further investigate the mechanistic basis of the effects triggered by pharmacological Cx hemichannel inhibition, whole transcriptome microarray analysis was performed on liver tissue of saline-treated, TAT-Gap24-treated, and TAT-Gap19-treated NASH mice. Several genes associated with fatty acid transport, inflammation and oxidative stress became differentially expressed upon Cx hemichannel inhibition. Of those genes, CD36, an important protein in the transport of fatty acids into cells, such as monocytes/macrophages, smooth muscle cells, Kupffer cells and stellate cells, was downregulated both at the RNA and protein level in TAT-Gap19-treated NASH animals. CD36 was recently described to be involved in NASH, as its expression is elevated in *ob/ob* mice, *db/db* mice and mice fed a high-fat diet, and highly correlates with hepatic triglyceride content^30^. Protein levels of NADPH oxidase were also downregulated in TAT-Gap19-treated NASH animals. NADPH oxidase was recently reported to have a malicious effect in NASH, since mice deficient in NADPH oxidase fed a high-fat diet are protected from developing NASH^45^. Other oxidative stress markers, such as *Gsta2* and *Hmox1*, which code for glutathione *S*-transferase and heme oxygenase, respectively, are often elevated in NAFLD^46^, but were decreased in TAT-Gap19-treated NASH animals. Interestingly, higher *Cyp7b1* gene expression was found in TAT-Gap19-treated NASH animals. CYP7B1 or oxysterol 7α-hydroxylase partially catalyzes the conversion of cholesterol to chenodeoxycholic acid, which could explain the reduced liver cholesterol levels in TAT-Gap19-treated NASH animals. In human NASH samples, CYP7B1 expression is increased and this is believed to be an attempt by the liver to reduce hepatotoxicity during disease progression to NASH^47^. Other negatively affected genes include *Adgre1* ^48^, *Cd5l* ^49^, and *Cd68* ^50^, all which are associated with NAFLD pathology. In fact, *Cd68* is a typical monocyte/macrophage marker^51^, which could suggest that TAT-Gap19 has an effect on the recruitment of monocytes to the liver^52^. However, it still needs to be determined whether these gene changes are a direct or indirect effect of the inhibition of Cx32 and Cx43 hemichannels^53^. It can not be ruled out that TAT-Gap19 also inhibits Cx43 hemichannels in other cell types, such as Kupffer cells or adipocytes. In case of the former, this would explain the decrease in levels of inflammatory cytokines, including IL-1β, TNF-β and IL-6^51^. Concerning adipocytes, a possible explanation could be presented for the decrease in relative fat weight. The same applies to Cx32, which is also expressed in cells of the stomach and intestines^54, 55^. In fact, involvement of the gut microbiome in the pathogenesis of NASH is increasingly accepted, since diet can modulate the gut microbiome and the subsequent inflammatory response^56^. As Cxs are key drivers of inflammation^57^, it seems plausible that Cx32 and/or Cx43 play a role in the liver-gut axis and thus in NASH. While the results of the current study show that Cx32 hemichannel inhibition alleviates pathological NASH features, whole-body knock-out of Cx32 in NASH mice was found to result in higher lipid content, inflammation and oxidative stress^15^. This could point to different roles for Cx32 gap junctions and Cx32 hemichannels in NASH, with the former fulfilling a protective function and the latter acting as pathological pores. It remains to be elucidated how exactly inhibition of Cx hemichannels can cause these effects in experimental NASH. Analysis of The Human Metabolome Database reveals that there are over 35000 biological molecules that could theoretically pass through gap junctions or Cx hemichannels^58^. There has been a large push in the last 20 years to determine at least some of the signaling molecules, ions and metabolites passing through specific gap junctions and Cx hemichannels^59^. Out of these signals, it is likely that ATP is one of the major players in the working mechanism of inhibition of Cx hemichannels, as it is associated with a diverse array of pathological processes, especially through purinergic signaling^60^. Purinergic receptors allow immune cells to recognize ATP released from damaged or stressed host cells. Thus, purinergic signaling of immune cells serves as an important function in the recognition of danger signals. Moreover, phagocytes recognize ATP that is released by stressed cells as a ‘find-me signal’, which guides phagocytes to inflammatory sites and promotes clearance of damaged and apoptotic cells^61^.

In summary, inhibition of Cx hemichannels may open perspectives for the establishment of new therapeutic strategies for NASH treatment.

## Materials and Methods

### Animals and treatment

8-week old male C57BL/6 mice were housed in the animal facility of the Department of Pathology, School of Veterinary Medicine and Animal Science of the University of São Paulo-Brazil. The animals were kept in a room with ventilation, relative humidity, controlled temperature and light/dark cycle 12:12, and were given water and a CHFD (35% total fat and 54% trans fatty enriched) (Rhoster, Brazil) or ND *ad libitum*. After 8 weeks of diet, an osmotic pump (Alzet, USA) was surgically implanted in the abdominal cavity, which ensured sustained release of 1 mg/kg/day TAT-Gap24 or TAT-Gap19 (>90% purity) (ThermoFischer, Germany) or saline for another 2 weeks, while continuing the diet. Because of persistent solubility issues, scrambled peptides have not been routinely included in these experiments. Mice were euthanized after 10 weeks by isoflurane-induced anaesthesia and exsanguination during sampling. Blood samples were drawn into heparinized syringes and centrifuged for 10 min at 4000 × *g*, and serum was stored at −80 °C. Liver and abdominal fat tissues of each animal were weighed, and relative liver and fat weights were calculated. Liver fragments were fixed in 10% phosphate-buffered formalin or snap-frozen in liquid nitrogen with storage at −80 °C. This study has been approved by the Committee on Bioethics of the School of Veterinary Medicine and Animal Science of the University of São Paulo (Protocol number 9999100314) and all animals received humane care according to the criteria outlined in the “Guide for the Care and Use of Laboratory Animals”.

### Hepatocyte isolation and cultivation

Male outbred Sprague-Dawley rats (Charles River Laboratories, Belgium) were kept under controlled environmental conditions with free access to food and water. Hepatocytes were isolated by use of a 2-step collagenase perfusion method, including purification by serial differential centrifugation, and cell viability was assessed by trypan blue exclusion^35^. Viable ( ≥85%) hepatocytes were plated at a density of 0.56 × 10^5^ cells per cm^2^ in William’s medium E (Invitrogen, USA) supplemented with 7 ng/mL glucagon, 292 mg/mL *L*-glutamine, antibiotics (7.33 IU of sodium benzyl penicillin, 50 μg/mL kanamycin monosulfate, 10 μg/mL sodium ampicillin, and 50 μg/ml streptomycin sulfate), and 10% fetal bovine serum. After 4 h, 24 h and 48 h, the cell culture medium was removed and replaced by serum-free medium supplemented with 25 μg/mL hydrocortisone sodium hemisuccinate and 0.5 μg/mL insulin. Procedures for the housing of rats, and isolation and cultivation of hepatocytes were approved by the Ethical Committee for Animal Experiments of the Vrije Universiteit Brussel (Project number 14-210-1) and all animals received humane care according to the criteria outlined in the “Guide for the Care and Use of Laboratory Animals”.

### Fluorescence recovery after photobleaching

For FRAP analysis 24 h after cell plating, cultured rat hepatocytes were exposed to 50 µM CBX, 20 µM TAT-Gap24, 20 µM TAT-Gap19 or vehicle control for 30 min, 24 h and 48 h. FRAP analysis was performed as previously described^37^. Fluorescence in the bleached cell was expressed as the percentage of recovery relative to the starting level just before photobleaching. A similar analysis was performed on cells distant from the bleached area to monitor photobleaching outside the targeted region. The recovery values for each experiment were normalized to the corresponding vehicle control condition. Additionally, Y_0_, plateau, tau, half-time and mobile fraction values were calculated following a non-linear curve fit.

### Measurement of extracellular adenosine triphosphate

Extracellular released ATP was measured using a commercial luciferin/luciferase assay kit (Sigma, USA) as previously described^37^. TAT-Gap24 and TAT-Gap19 were first incubated at 37 °C for 0 min, 6 days or 20 days in a classic incubator (Galaxy 170S, New Brunswick, Germany). Primary rat hepatocyte cultures were then exposed to 20 µM TAT-Gap24, 20 µM TAT-Gap19, 50 µM CBX or vehicle control for 30 min. These experiments were performed 24 h after isolation of the primary hepatocytes.

### Histopathological examination

Liver tissue samples were fixed in 10% phosphate-buffered formalin for 24 h and embedded in paraffin wax. Samples were cut into 5 µm sections and stained with hematoxylin-eosin for evaluation of steatosis, hepatocellular ballooning, lobular inflammation and NAS as previously described^22^. The degree of steatosis was graded using the following 4-point scale, namely grade 0, steatosis involving <5% of hepatocytes; grade 1, steatosis involving up to 33% of hepatocytes; grade 2, steatosis involving 33–66% of hepatocytes; grade 3, steatosis involving >66% of hepatocytes. Lobular inflammation was also graded on a 4-point scale, namely grade 0, no foci; grade 1, fewer than 2 foci per ×20 field; grade 2, 2 to 4 foci per ×20 field; grade 3, more than 4 foci per ×20 field. Hepatocyte ballooning was graded on a 3-point scale: 0, none; 1, a few balloon cells; 2, any/prominent balloon cells. For NAS, features of steatosis, lobular inflammation and hepatocyte ballooning were combined with values from 0 to 8.

### Analysis of serum aminotransaminases, serum and liver triglycerides and cholesterol

Liver lipids were extracted by means of chloroform/methanol. Specifically, liver tissue was homogenized in 1 mL 2:1 chloroform/methanol solution and shaken for 1 h in a Thermomixer (Eppendorf, Germany) at 22 °C. Samples were centrifuged for 10 min at 5000 × *g* after which the supernatant was taken. Next, 200 µL distilled water was added and samples were centrifuged for 5 min at 8000 × *g*. The bottom phase was dried overnight at 37 °C. *Prior* analysis, lipids were dissolved in 400 µL butanol (Sigma, USA). ALT, AST, triglycerides and cholesterol were measured by a chemistry analyser (Labmax 240, Labtest, Brazil). Results were expressed in IU/L for AST and ALT, mg/dL for serum triglycerides and cholesterol, and µg/mg for liver triglycerides and cholesterol.

### Analysis of liver inflammatory cytokines

Liver tissue was homogenized in lysis buffer with protease inhibitors (Roche, Germany). Homogenates were centrifuged at 14000 × g for 15 min at 4 °C and protein concentrations in supernatants were determined by the Bradford procedure^62^ using a commercial kit (Bio-Rad, USA) with bovine serum albumin as a standard. ELISA kits were used to measure levels of IL-1β, IL-6, IL-10, IFN-γ and TNF-α (BD Biosciences, USA) in liver^63^.

### Analysis of liver anti-oxidative enzymes

SOD activity in liver homogenates was determined using a microassay as described previously^64^. The SOD scavenging of superoxide anion radical was calculated by interpolation of the percentage of inhibition of the formazan formation using linear regression prepared with SOD from bovine erythrocytes (Sigma, USA). Determination of GPx activity was performed using the procedure as outlined previously^65^. Tert-butylhydroperoxide was used as a substrate and the formation of oxidized glutathione was monitored spectrophotometrically through nicotinamide adenine dinucleotide phosphate consumption at 340 nm over 4 min at 37 °C. GPx activity was calculated by linear regression using the percentage of inhibition promoted by GPx (Sigma, USA). Determination of GR activity was performed as previously described^66^. GR activity was calculated by linear regression using the percentage of inhibition of nicotinamide adenine dinucleotide phosphate oxidation promoted by GR (Sigma, USA). Catalase activity was determined as previously described^67^. Liver homogenate containing 0.05 µg/µL of protein (20 µL) was added to a microplate with 140 µL phosphate-buffered saline (50 mM with 0.1 mM ethylenediamine tetra-acetic acid, pH 7.4) and 40 µL 30 mM freshly prepared hydrogen peroxide. The absorbance was continuously monitored over 8 min at 30 °C at 240 nm. A standard curve was prepared using catalase enzyme (Sigma, USA). All results were expressed as U/mg protein.

### Whole transcriptome analysis

Total RNA was extracted from liver fragments using a GenElute Mammalian Total RNA Purification Miniprep Kit (Sigma, USA) according to the manufacturer’s instructions. Purity and quantification of the isolated RNA were determined by spectrophotometric analysis using a Nanodrop spectrophotometer (Thermo Scientific, USA). RNA integrity was analyzed by microfluidic analysis by means of an Agilent 2100 Bioanalyser (Agilent Technologies, USA). As such, 100 ng total RNA per sample was amplified with a Genechip 3′ IVT Express Kit, thereby following the manufacturer’s instructions (Affymetrix, Germany). Amplified RNA was purified with magnetic beads and 15 mg biotin-amplified RNA was treated with the fragmentation reagent. Subsequently, 12.5 µg fragmented amplified RNA was hybridized to Affymetrix Clariom™ D mouse arrays and placed in a Genechip Hybridization Oven-645 (Affymetrix, Germany) rotating at 60 rpm at 45 °C for 16 h. Thereafter, the arrays were washed on a Genechip Fluidics Station-450 (Affymetrix, Germany) and stained with an Affymetrix HWS kit. The chips were scanned with an Affymetrix Gene-Chip Scanner-3000-7G and quality control matrices were confirmed with Affymetrix GCOS software following the manufacturer’s guidelines. Background correction, summarization and normalization of all data were done with Expression Console and Affymetrix Transcriptome Analysis Console Software.

### Immunoblot analysis

Immunoblot analysis of liver tissue was performed as previously described^63^. Nitrocellulose/PVDF membranes were incubated overnight at 4 °C with primary antibody directed to Cx26 (1/1000 dilution), Cx32 (1/1000 dilution), Cx43 (1/1000 dilution), CD36 (1/250 dilution), NADPH oxidase (1/250 dilution) and LY86 (1/500 dilution) (Sigma, Belgium) followed by incubation for 1 h at room temperature with appropriate secondary antibody (1/1000 dilution for Cx26, Cx32 and Cx43; 1/500 dilution for CD36, NADPH oxidase and LY86) (Dako, Denmark). Densitometric analysis was performed using Image Lab 5.0 software (Bio-Rad, USA). For semi-quantification purposes, signals were normalized against total protein, measured by Stain-Free gels, and expressed as relative alterations compared to ND-fed animals.

### Statistical analysis

The number of biological (n) and technical (N) repeats for each analysis varied and is specified in the discussion of the results. All data were expressed as mean ± standard error of mean (SEM). Results were statistically processed by 1-way analysis of variance with *post hoc* Bonferroni correction. All data were processed using GraphPad Prism6 software, with probability (p) values of less than 0.05 considered as significant.

## Electronic supplementary material

Supplementary data
