# Supplementary material for: Inhibition of connexin hemichannels alleviates non-alcoholic steatohepatitis in mice
Source: Sci Rep. 2017 Aug 15;7:8268. doi: 10.1038/s41598-017-08583-w (PMC5557827; doi:10.1038/s41598-017-08583-w)

Inhibition of connexin hemichannels alleviates non-alcoholic steatohepatitis in mice

Joost Willebrords[a,$], Bruno Cogliati[b,$], Isabel Veloso Alves Pereira[b], Tereza Cristina da Silva[b], Sara Crespo Yanguas[a], Michaël Maes[a], Veronica Mollica Govoni[b], Andressa Lima[b], Daniele Aparecida Felisbino[b], Elke Decrock[c], Marina Sayuri Nogueira[d], Inar Alves de Castro[d], Isabelle Leclercq[e], Luc Leybaert[c], Robim Marcelino Rodrigues[a] and Mathieu Vinken[a,]*

[a]Department of *In Vitro* Toxicology and Dermato-Cosmetology, Faculty of Medicine and Pharmacy, Vrije Universiteit Brussel, Laarbeeklaan 103, 1090 Brussels, Belgium.

Joost Willebrords: E-mail: joost.willebrords@vub.ac.be; + Tel: 32 2 477 45 87

Sara Crespo Yanguas: E-mail: sara.crespo.yanguas@vub.ac.be; Tel: +32 2 477 45 87

Michaël Maes: E-mail: michael.mc.maes@vub.ac.be; Tel: +32 2 477 45 87

Robim Marcelino Rodrigues: rmarceli@vub.ac.be; Tel: +32 2 477 45 17

Mathieu Vinken: E-mail: mvinken@vub.ac.be; Tel: +32 2 477 45 87

[b]Department of Pathology, School of Veterinary Medicine and Animal Science, University of São Paulo, Av. Prof. Dr. Orlando Marques de Paiva 87, 05508-270, São Paulo, Brazil.

Bruno Cogliati E-mail: bcogliati@usp.br; Tel: +55 11 30 91 12 00

Isabel Veloso Alves Pereira: isabelveloso@gmail.com; Tel: +55 11 30 91 12 00

Tereza Cristina da Silva: terezacs@usp.br; Tel: +55 11 30 91 12 00

Veronica Mollica Govoni: veronica.mgovoni@gmail.com; Tel: +55 11 30 91 12 00

Andressa Lima: andressalima8282@gmail.com; Tel: +55 11 30 91 12 00

Daniele Aparecida Felisbino: dannoza@hotmail.com; Tel: +55 11 30 91 12 00

[c]Department of Basic Medical Sciences, Physiology Group, Ghent University, De Pintelaan 185, 9000 Ghent, Belgium
Elke Decrock: E-mail: elke.decrock@ugent.be; Tel: +32 9 332 39 73
Luc Leybaert: E-mail: luc.leybaert@ugent.be; Tel: +32 9 332 33 66

[d]Department of Food and Experimental Nutrition, Faculty of Pharmaceutical Sciences, University of São Paulo, Av. Prof. Lineu Prestes 580, 05508-270 São Paulo, Brazil.
Marina Sayuri Nogueira: masayuri.nogueira@gmail.com; Tel: +55 11 30 91 36 30
Inar Alves de Castro: inar@usp.br; Tel: +55 11 30 91 36 30

[e]Laboratory of hepatogastroenterology, Institut de Recherche Expérimentale et clinique, Université catholique de Louvain, Avenue Mounier 53, 1200 Brussels, Belgium.
Isabelle Lecercq: isabelle.leclercq@uclouvain.be, Tel: +32 2 764 52 73

$ Equally contributing first authors.
* Corresponding author: Mathieu Vinken, Laarbeeklaan 103, 1090 Brussels, Belgium, E-mail: mvinken@vub.ac.be, Tel: +32 2 477 45 87.

Supplementary Method 1. Reverse transcription quantitative real-time polymerase chain reaction. After RNA extraction, 2 µg RNA was reversely transcribed into cDNA with an iScript™ cDNA Synthesis Kit (Bio-Rad, USA) using an iCycler iQ™ (Bio-Rad, USA) followed by cDNA purification using a GenElute™ PCR Clean-Up Kit. cDNA products were quantitatively amplified using Taqman probes and primers (Applied Biosystems, USA) targeted toward *Gja1* (Cx43), *Gjb1* (Cx32) and *Gjb2* (Cx26) and candidate reference genes (**Supplementary Table 4**). All samples were analyzed in duplicate. Each run included a serial dilution of a pooled cDNA mix from all cDNA samples and 2 no template controls to estimate the quantitative polymerase chain reaction efficiency. For reverse transcription quantitative real-time polymerase chain reaction (RT-qPCR) analysis, a reaction mix was prepared containing TaqMan® Fast Advanced Master Mix (Applied Biosystems, USA), Assay-on-Demand™ Gene Expression Assay Mix (Applied Biosystems, USA) and cDNA diluted in nuclease-free water. The qPCR conditions, using a StepOnePlus™ real-time PCR system (Life Technologies, USA), included incubation for 20 s at 95°C followed by 40 cycles of denaturation for 1 s at 95°C and annealing for 20 s at 60°C. Efficiency was estimated by StepOne Plus™ system's software and only data with PCR efficiency between 90% and 110% were used. Stable candidate reference genes for normalization purposes were identified out of a pool of 6 genes as determined by geNorm using the qbase^{+} software (Biogazelle, Belgium). The resulting ΔCq values of the test samples were normalized to those of the calibrator samples, yielding ΔΔCq values. Relative alterations (fold change) in RNA levels were calculated according to the Livak $2^{-\Delta\Delta Cq}$ formula [26].

Supplementary Method 2. Cytotoxicity assay. Cell viability after 24 h and 48 h of treatment with 0, 50, 100, 250 and 500 µM TAT-Gap24 or TAT-Gap19 was assayed through a 3-(4,5-dimethylthiazol-2-yl)-2,5-diphenyltetrazolium bromide (MTT) test. Specifically, medium was removed from culture plates, followed by incubation for 1.5 h with MTT (Sigma Aldrich, USA), solubilized in hepatocyte culture medium. Subsequently, MTT solution was removed and replaced by an isopropanol (Thermo Scientific, USA)/1 M HCl 20:1 solution. Culture plates were shaken for 10 min and absorbance was measured at 570 nm using a spectrophotometer (Victor®, Perkin Elmer Precisely, USA). Results were normalized to 100%, which corresponds with values at 0 µM.

Supplementary Table 1. Differentially expressed genes in liver tissue of CHFD-fed mice. RNA was extracted from liver tissue of CHFD-fed mice and ND-fed mice, and whole transcriptome microarray analysis was performed using Affymetrix Clariom™ D mouse arrays. Background correction, summarization and normalization of all data were done with Expression Console and Affymetrix Transcriptome Analysis Console Software. Fold change threshold was set at <-1.5 and >1.5, and results were statistically processed by 1-way analysis of variance.

Gene symbol	Fold change	p-value	Description
Cyp4a14	11,30	0,000099	cytochrome P450, family 4, subfamily a, polypeptide 14
Snora62	6,36	0,000000	small nucleolar RNA, H/ACA box 62
Mir505	4,35	0,000000	microRNA 505
Neat1	3,44	0,000000	nuclear paraspeckle assembly transcript 1 (non-protein coding)
Snora75	3,42	0,000274	small nucleolar RNA, H/ACA box 75
Scarna17	3,38	0,000000	small Cajal body-specific RNA 17
Gtpbp4-ps1	3,06	0,000677	GTP binding protein 4, pseudogene 1 [Source:MGI Symbol;Acc:MGI:5521100]
Mir5125	3,02	0,000000	microRNA 5125
Mir122	2,92	0,000361	microRNA 122
Egln3	2,82	0,000147	EGL nine homolog 3 (C. elegans), mRNA (cDNA clone MGC:36685 IMAGE:5371854), complete cds.
Mir5124a; Mir5124	2,69	0,000013	microRNA 5124a [Source:MGI Symbol;Acc:MGI:4950449]; microRNA 5124
Serpina7	2,61	0,000823	serine (or cysteine) peptidase inhibitor, clade A (alpha-1 antiproteinase, antitrypsin), member 7
Snord55	2,40	0,000000	small nucleolar RNA, C/D box 55
Snord35a	2,38	0,000000	small nucleolar RNA, C/D box 35A
Snord123	2,38	0,000000	small nucleolar RNA, C/D box 123
Malat1; MALAT1	2,37	0,000001	metastasis associated lung adenocarcinoma transcript 1 (non-coding RNA); Metastasis associated lung adenocarcinoma transcript 1
Mir1948	2,34	0,000121	microRNA 1948
4632427E13Rik	2,33	0,000000	RIKEN cDNA 4632427E13 gene
LOC432823	2,33	0,000000	similar to hypothetical protein MGC37588, mRNA (cDNA clone MGC:28125 IMAGE:3980327), complete cds.
LOC432823	2,28	0,000007	similar to hypothetical protein MGC37588, mRNA (cDNA clone MGC:28125 IMAGE:3980327), complete cds.
Vnn1	2,23	0,000001	vanin 1
Snord14e	2,21	0,000000	small nucleolar RNA, C/D box 14E
Ifi27l2b	2,20	0,000011	interferon, alpha-inducible protein 27 like 2B
Lcn2	2,17	0,008407	lipocalin 2
Apoa4	2,17	0,000348	apolipoprotein A-IV
Snord104	2,17	0,000000	small nucleolar RNA, C/D box 104
Mir5123	2,15	0,001749	microRNA 5123
Mmp12	2,15	0,000000	matrix metallopeptidase 12
Igfbp1	2,13	0,016793	insulin-like growth factor binding protein 1
Lyz2	2,11	0,000001	lysozyme 2
Igkv4-55	2,10	0,000024	immunoglobulin kappa variable 4-55
Snord11	2,09	0,000068	small nucleolar RNA, C/D box 11

Snord70	2,09	0,000014	small nucleolar RNA, C/D box 70
Mir5125	2,08	0,000000	microRNA 5125
Snord82	2,07	0,000001	small nucleolar RNA, C/D box 82
C530030P08Rik	2,07	0,000005	RIKEN cDNA C530030P08 gene
Drr1	2,05	0,000000	developmentally regulated repeat element-containing transcript 1
LOC432823	2,00	0,000001	similar to hypothetical protein MGC37588, mRNA (cDNA clone MGC:28125 IMAGE:3980327), complete cds.
Slc25a25	1,99	0,001893	solute carrier family 25 (mitochondrial carrier, phosphate carrier), member 25
Rn4.5s	1,99	0,000041	4.5S RNA; 4.5S RNA (Rn4.5s), ribosomal RNA.
Gpnmb	1,98	0,000000	glycoprotein (transmembrane) nmb
Cd36	1,97	0,000026	CD36 antigen
Lgals1	1,95	0,000001	lectin, galactose binding, soluble 1
Lilrb4a	1,94	0,000001	leukocyte immunoglobulin-like receptor, subfamily B, member 4A
Snord42a	1,94	0,000001	small nucleolar RNA, C/D box 42A
Mir297-1	1,93	0,000002	microRNA 297-1
Gtpbp4-ps4	1,91	0,000699	GTP binding protein 4, pseudogene 4 [Source:MGI Symbol;Acc:MGI:5521103]
Lilr4b	1,91	0,000004	leukocyte immunoglobulin-like receptor, subfamily B, member 4B
S100a8	1,90	0,000786	S100 calcium binding protein A8 (calgranulin A)
S100a11	1,88	0,000946	S100 calcium binding protein A11; S100 calcium binding protein A11 (calgizzarin)
LOC432823	1,88	0,000001	similar to hypothetical protein MGC37588, mRNA (cDNA clone MGC:28125 IMAGE:3980327), complete cds.
Lyz1	1,88	0,000003	lysozyme 1
Snord35b	1,86	0,000000	small nucleolar RNA, C/D box 35B
Hamp	1,84	0,000574	hepcidin antimicrobial peptide
Cd68	1,83	0,000002	CD68 antigen (Cd68), mRNA.; CD68 antigen
Mirlet7d	1,83	0,000000	microRNA let7d
Clec7a	1,81	0,000339	C-type lectin domain family 7, member a
Marco	1,81	0,001656	macrophage receptor with collagenous structure
Lncpint	1,80	0,000001	long non-protein coding RNA, Trp53 induced transcript
Adam4	1,79	0,000006	a disintegrin and metallopeptidase domain 4
2010003K11Rik	1,79	0,001104	RIKEN cDNA 2010003K11 gene
Mir297a-2	1,78	0,000027	microRNA 297a-2
Zfp36l1-ps	1,78	0,000025	zinc finger protein 36, C3H type-like 1, pseudogene
Mir466f-3	1,78	0,000076	microRNA 466f-3
Zfp36l1	1,77	0,000000	zinc finger protein 36, C3H type-like 1
Mir6983	1,77	0,000064	microRNA 6983
Snord110	1,75	0,000346	small nucleolar RNA, C/D box 110
Hamp2	1,75	0,001496	hepcidin antimicrobial peptide 2
G0s2	1,74	0,002499	G0/G1 switch gene 2
LOC102637418	1,74	0,000012	protein FAM205A-like
Rny1	1,73	0,000001	RNA, Y1 small cytoplasmic, Ro-associated
Rnu11	1,72	0,000008	U11 small nuclear RNA
Mir7026	1,71	0,000163	microRNA 7026
Snord49a	1,71	0,000055	small nucleolar RNA, C/D box 49A
Mir1933	1,70	0,000240	microRNA 1933
Igkv4-62	1,69	0,000032	immunoglobulin kappa variable 4-62
Abcd2	1,69	0,009070	ATP-binding cassette, sub-family D (ALD), member 2
4930405O22Rik	1,69	0,000004	RIKEN cDNA 4930405O22 gene
Mir1191	1,68	0,000006	microRNA 1191
Snord8	1,67	0,000000	small nucleolar RNA, C/D box 8
Snord83b	1,67	0,000006	small nucleolar RNA, C/D box 83B
Igkv4-80	1,66	0,000176	immunoglobulin kappa variable 4-80

Igkv4-79	1,66	0,000076	immunoglobulin kappa variable 4-79
Tubb2a	1,66	0,001963	tubulin, beta 2A class IIA
Mir467c	1,65	0,010498	microRNA 467c
Fabp2	1,65	0,000001	fatty acid binding protein 2, intestinal
Rny1	1,65	0,000005	RNA, Y1 small cytoplasmic, Ro-associated
Igkv4-68	1,65	0,000332	immunoglobulin kappa variable 4-68
Mir669h	1,65	0,000018	microRNA 669h
Slc7a7	1,65	0,000025	solute carrier family 7 (cationic amino acid transporter, y+ system), member 7
Ctss	1,63	0,000000	cathepsin S; cathepsin S (Ctss), transcript variant 1, mRNA.
Peg10	1,63	0,000029	paternally expressed 10; Synthetic construct Mus musculus clone IMAGE:100062263, MGC:190439 paternally expressed 10 (Peg10) mRNA, encodes complete protein.
Ighv1-64	1,63	0,003653	immunoglobulin heavy variable 1-64
Mir6353	1,62	0,000074	microRNA 6353
Chka	1,62	0,001245	choline kinase alpha; choline kinase alpha (Chka), transcript variant 2, mRNA.; choline kinase alpha (Chka), transcript variant 1, mRNA.
Snord73a	1,61	0,000083	small nucleolar RNA, C/D box U73A
Ighv1-55	1,61	0,000298	immunoglobulin heavy variable 1-55
Mir1192	1,61	0,000144	microRNA 1192
Pten	1,61	0,000006	phosphatase and tensin homolog
Acnat2	1,60	0,001981	acyl-coenzyme A amino acid N-acyltransferase 2
Mir292b	1,60	0,003407	microRNA 292b
Gtpbp4-ps3	1,60	0,000090	GTP binding protein 4, pseudogene 3 [Source:MGI Symbol;Acc:MGI:5521093]
Snord71	1,60	0,000017	small nucleolar RNA, C/D box 71
Plin4	1,60	0,000001	perilipin 4
G6pc	1,59	0,035526	glucose-6-phosphatase, catalytic
4930423O20Rik	1,59	0,000106	RIKEN cDNA 4930423O20 gene
Spon2	1,58	0,000113	spondin 2, extracellular matrix protein; spondin 2, extracellular matrix protein (Spon2), mRNA.
Igkv4-59	1,58	0,000049	immunoglobulin kappa variable 4-59
Igkv4-57	1,58	0,000088	immunoglobulin kappa variable 4-57
Scarna9	1,58	0,014807	small Cajal body-specific RNA 9
Ogt	1,58	0,000000	O-linked N-acetylglucosamine (GlcNAc) transferase (UDP-N-acetylglucosamine:polypeptide-N-acetylglucosaminyl transferase); O-linked N-acetylglucosamine (GlcNAc) transferase (UDP-N-acetylglucosamine:polypeptide-N-acetylglucosaminyl transferase) (Ogt), mRNA.
Mir680-3	1,58	0,000001	microRNA 680-3
Cd74; Mir5107	1,58	0,006169	CD74 antigen (invariant polypeptide of major histocompatibility complex, class II antigen-associated); microRNA 5107
2210017G18Rik	1,57	0,000253	RIKEN cDNA 2210017G18 gene
Igkv4-54	1,57	0,000024	immunoglobulin kappa chain variable 4-54
Erdr1	1,57	0,000033	erythroid differentiation regulator 1
Mir6920	1,57	0,002315	microRNA 6920
Snord89	1,56	0,000152	small nucleolar RNA, C/D box 89
Fcer1g	1,56	0,000144	Fc receptor, IgE, high affinity I, gamma polypeptide
Rhoc	1,56	0,000000	ras homolog gene family, member C
C3ar1	1,56	0,000003	complement component 3a receptor 1
Ighv1-26	1,56	0,000113	immunoglobulin heavy variable 1-26
Mir3471-1	1,55	0,000380	microRNA 3471-1
Igkv4-72	1,55	0,000049	immunoglobulin kappa chain variable 4-72
Ccnd1; Mir3962	1,55	0,000002	cyclin D1; microRNA 3962
Snora47	1,55	0,000002	small nucleolar RNA, H/ACA box 47
Lpl	1,54	0,000001	lipoprotein lipase; lipoprotein lipase (Lpl), mRNA.

Mir467h	1,54	0,000182	microRNA 467h [Source:MGI Symbol;Acc:MGI:3783381]
Dusp6	1,54	0,049192	dual specificity phosphatase 6
Mir325	1,54	0,008092	microRNA 325
Mir7213	1,54	0,000010	microRNA 7213
Tbrg3	1,54	0,000004	transforming growth factor beta regulated gene 3; transforming growth factor beta regulated gene 3 (Tbrg3), non-coding RNA.
Mir466f-4	1,53	0,000005	microRNA 466f-4 [Source:MGI Symbol;Acc:MGI:3783374]
Cd84	1,53	0,000000	CD84 antigen
Clk1	1,53	0,000000	CDC-like kinase 1; Synthetic construct Mus musculus clone IMAGE:100064101, MGC:193472 CDC-like kinase 1 (Clk1) mRNA, encodes complete protein.; CDC-like kinase 1 (Clk1), transcript variant 2, non-coding RNA.
Gpr52	1,53	0,001311	G protein-coupled receptor 52; G protein-coupled receptor 52 (Gpr52), mRNA.
Cd5l	1,53	0,000814	CD5 antigen-like
Arntl	1,53	0,001270	aryl hydrocarbon receptor nuclear translocator-like
Mir540	1,53	0,000301	microRNA 540
Mir5623	1,53	0,000022	microRNA 5623
9030622O22Rik; RP23-207P16.1	1,52	0,000585	RIKEN cDNA 9030622O22 gene [Source:MGI Symbol;Acc:MGI:1918820]; novel transcript
C1qc	1,52	0,000047	complement component 1, q subcomponent, C chain
St5	1,52	0,001403	suppression of tumorigenicity 5
Snord14a	1,52	0,000189	small nucleolar RNA, C/D box 14A
Mir15a	1,51	0,000518	microRNA 15a
Snord87	1,51	0,000045	small nucleolar RNA, C/D box 87
Ifi30	1,51	0,000001	interferon gamma inducible protein 30
Elovl5	1,51	0,002764	ELOVL family member 5, elongation of long chain fatty acids (yeast)
Paqr9	1,51	0,001179	progestin and adipoQ receptor family member IX
Slc17a8	1,51	0,000283	solute carrier family 17 (sodium-dependent inorganic phosphate cotransporter), member 8
Mir5099	1,51	0,000017	microRNA 5099
Ighv1-53	1,51	0,001465	immunoglobulin heavy variable 1-53
Snord66	1,51	0,010138	small nucleolar RNA, C/D box 66
E030024N20Rik	1,51	0,000581	peptidylprolyl isomerase A pseudogene 8
Pisd-ps3	1,51	0,000000	phosphatidylserine decarboxylase, pseudogene 3
Mir5099	1,51	0,000017	microRNA 5099
Snora73a	1,51	0,000655	small nucleolar RNA, H/ACA box 73a
Lgr4	-1,51	0,000012	leucine-rich repeat-containing G protein-coupled receptor 4; Synthetic construct Mus musculus clone IMAGE:100064037, MGC:193408 leucine-rich repeat-containing G protein-coupled receptor 4 (Lgr4) mRNA, encodes complete protein.
Gatc	-1,51	0,000221	glutamyl-tRNA(Gln) amidotransferase, subunit C; glutamyl-tRNA(Gln) amidotransferase, subunit C homolog (bacterial)
Cyp2f2	-1,51	0,000004	cytochrome P450, family 2, subfamily f, polypeptide 2
Oat-rs1	-1,51	0,000022	ornithine aminotransferase related sequence 1
Nr1d2	-1,51	0,000003	nuclear receptor subfamily 1, group D, member 2
Amacr	-1,51	0,000001	alpha-methylacyl-CoA racemase
Cmbl	-1,51	0,000000	carboxymethylenebutenolidase-like (Pseudomonas)
Cyp2c54	-1,51	0,000822	cytochrome P450, family 2, subfamily c, polypeptide 54; Synthetic construct Mus musculus clone IMAGE:100066250, MGC:195387 cytochrome P450, family 2, subfamily c, polypeptide 54 (Cyp2c54) mRNA, encodes complete protein.
Slc43a1	-1,52	0,000000	solute carrier family 43, member 1
Neb	-1,52	0,000134	nebulin
Dbt	-1,52	0,000005	dihydrolipoamide branched chain transacylase E2
Il6ra	-1,52	0,000062	interleukin 6 receptor, alpha
Cyp4a12b	-1,52	0,000882	cytochrome P450, family 4, subfamily a, polypeptide 12B

Ugt2b38	-1,52	0,000000	UDP glucuronosyltransferase 2 family, polypeptide B38
Vps33b	-1,52	0,000214	vacuolar protein sorting 33B (yeast)
Slc27a5	-1,52	0,000001	solute carrier family 27 (fatty acid transporter), member 5
Scap	-1,52	0,000013	SREBF chaperone; SREBF chaperone (Scap), transcript variant 2, mRNA.
Snx12	-1,52	0,000038	sorting nexin 12
Snord118	-1,52	0,019691	small nucleolar RNA, C/D box 118
Manea	-1,53	0,000019	mannosidase, endo-alpha
Sult1d1	-1,53	0,000006	sulfotransferase family 1D, member 1
Slc25a15	-1,53	0,000007	solute carrier family 25 (mitochondrial carrier ornithine transporter), member 15
DLEU2_5	-1,53	0,000718	Deleted in lymphocytic leukemia 2 conserved region 5
Prodh	-1,53	0,000003	proline dehydrogenase
Fabp5l2	-1,53	0,001881	fatty acid binding protein 5-like 2 [Source:MGI Symbol;Acc:MGI:3644282]
Fabp5	-1,54	0,007974	fatty acid binding protein 5, epidermal
mt-Tn	-1,54	0,006425	mitochondrially encoded tRNA asparagine [Source:MGI Symbol;Acc:MGI:102479]
Atp6v0a1	-1,54	0,000000	ATPase, H+ transporting, lysosomal V0 subunit A1
Ttc38	-1,54	0,000002	tetratricopeptide repeat domain 38
Myeov2	-1,55	0,000005	myeloma overexpressed 2
Mfsd1	-1,55	0,000007	major facilitator superfamily domain containing 1
Ap1ar	-1,55	0,000054	adaptor-related protein complex 1 associated regulatory protein
Slc25a13	-1,55	0,000000	solute carrier family 25 (mitochondrial carrier, adenine nucleotide translocator), member 13
F7	-1,55	0,000004	coagulation factor VII
Gstt3	-1,55	0,000006	glutathione S-transferase, theta 3
Slc25a21	-1,55	0,000002	solute carrier family 25 (mitochondrial oxodicarboxylate carrier), member 21
Cmah	-1,55	0,000023	cytidine monophospho-N-acetylneuraminic acid hydroxylase; cytidine monophospho-N-acetylneuraminic acid hydroxylase (Cmah), transcript variant 2, mRNA.; cytidine monophospho-N-acetylneuraminic acid hydroxylase (Cmah), transcript variant 1, mRNA.
Zfp960	-1,55	0,000003	zinc finger protein 960
Pank1	-1,55	0,000000	pantothenate kinase 1
Ly96	-1,56	0,000623	lymphocyte antigen 96
Cyp2u1	-1,56	0,000007	cytochrome P450, family 2, subfamily u, polypeptide 1; Synthetic construct Mus musculus clone IMAGE:100016290, MGC:184492 cytochrome P450, family 2, subfamily u, polypeptide 1 (Cyp2u1) mRNA, encodes complete protein.
Bhlhe41	-1,56	0,000114	basic helix-loop-helix family, member e41; Synthetic construct Mus musculus clone IMAGE:100069535, MGC:199437 basic helix-loop-helix family, member e41 (Bhlhe41) mRNA, encodes complete protein.
Prss8	-1,56	0,000003	protease, serine 8 (prostasin); protease, serine, 8 (prostasin); protease, serine, 8 (prostasin) (Prss8), mRNA.
Ccl9	-1,56	0,000005	chemokine (C-C motif) ligand 9
Mia2	-1,56	0,000009	melanoma inhibitory activity 2; Synthetic construct Mus musculus clone IMAGE:100014983, MGC:180329 melanoma inhibitory activity 2 (Mia2) mRNA, encodes complete protein.
Rps2-ps6	-1,56	0,000006	ribosomal protein S2, pseudogene 6 [Source:MGI Symbol;Acc:MGI:3644876]
D15Ertd621e	-1,56	0,000007	DNA segment, Chr 15, ERATO Doi 621, expressed
March6	-1,56	0,000003	membrane-associated ring finger (C3HC4) 6
Snx29	-1,56	0,000002	sorting nexin 29
Sgms2	-1,57	0,000032	sphingomyelin synthase 2
Dhdh	-1,57	0,000013	dihydrodiol dehydrogenase (dimeric)
Wdr46	-1,57	0,000000	WD repeat domain 46
C8b	-1,58	0,000008	complement component 8, beta polypeptide

Mir135a-1	-1,58	0,000003	microRNA 135a-1
Acp1	-1,58	0,000000	acid phosphatase 1, soluble
Ggact	-1,58	0,000000	gamma-glutamylamine cyclotransferase
Abat	-1,58	0,000002	4-aminobutyrate aminotransferase
Srd5a2	-1,58	0,000333	steroid 5 alpha-reductase 2
Snora34; Mir1291	-1,59	0,003544	small nucleolar RNA, H/ACA box 34; microRNA 1291
Alg14	-1,59	0,000046	asparagine-linked glycosylation 14
Sephs2	-1,59	0,000001	selenophosphate synthetase 2
Rpl15-ps2	-1,59	0,000034	ribosomal protein L15, pseudogene 2
Commd4	-1,59	0,000001	COMM domain containing 4
0610007P14Rik	-1,59	0,000000	RIKEN cDNA 0610007P14 gene
Snai2	-1,59	0,000072	snail family zinc finger 2; snail homolog 2 (Drosophila)
Jmjd8	-1,59	0,000000	jumonji domain containing 8
Upp2	-1,60	0,000256	uridine phosphorylase 2
Hsd3b5	-1,60	0,003665	hydroxy-delta-5-steroid dehydrogenase, 3 beta- and steroid delta-isomerase 5
Dnase2b	-1,60	0,000003	deoxyribonuclease II beta
Agmat	-1,60	0,000001	agmatine ureohydrolase (agmatinase)
Aldh1l1	-1,60	0,000002	aldehyde dehydrogenase 1 family, member L1
Tspan12	-1,60	0,000015	tetraspanin 12
Cyp2a5; Cyp2a4	-1,60	0,003458	cytochrome P450, family 2, subfamily a, polypeptide 5; cytochrome P450, family 2, subfamily a, polypeptide 4
Ehd3	-1,60	0,000006	EH-domain containing 3
As3mt	-1,60	0,000135	arsenic (+3 oxidation state) methyltransferase
Cd59a	-1,61	0,000023	CD59a antigen
Rarres1	-1,61	0,000377	retinoic acid receptor responder (tazarotene induced) 1
Dbp	-1,61	0,000311	D site albumin promoter binding protein
Slc16a10	-1,61	0,000000	solute carrier family 16 (monocarboxylic acid transporters), member 10
Tmem97	-1,61	0,000000	transmembrane protein 97
Abca6	-1,61	0,000001	ATP-binding cassette, sub-family A (ABC1), member 6
Ugt3a1	-1,61	0,000003	UDP glycosyltransferases 3 family, polypeptide A1
Pla1a	-1,61	0,000000	phospholipase A1 member A
Wee1	-1,62	0,000010	WEE 1 homolog 1 (S. pombe)
Ldlr	-1,62	0,000000	low density lipoprotein receptor
Xlr3d-ps	-1,62	0,016325	X-linked lymphocyte-regulated 3D, pseudogene
Igfals	-1,62	0,000020	insulin-like growth factor binding protein, acid labile subunit
Sfxn2	-1,62	0,000001	sideroflexin 2
Idh3b	-1,63	0,000002	isocitrate dehydrogenase 3 (NAD+) beta
Cth	-1,63	0,000000	cystathionase (cystathionine gamma-lyase)
Vma21-ps	-1,63	0,000036	VMA21 vacuolar H+-ATPase homolog (S. cerevisiae), pseudogene [Source:MGI Symbol;Acc:MGI:3643145]
Saa4	-1,63	0,000238	serum amyloid A 4
Camk1d	-1,64	0,000002	calcium/calmodulin-dependent protein kinase ID
Mmd2	-1,64	0,000208	monocyte to macrophage differentiation-associated 2
Gys2	-1,64	0,000001	glycogen synthase 2
Got1	-1,64	0,000608	glutamic-oxaloacetic transaminase 1, soluble; glutamate oxaloacetate transaminase 1, soluble
Cml2	-1,65	0,000001	camello-like 2
Ces3b	-1,65	0,000006	carboxylesterase 3B
Tmem184c	-1,65	0,000015	transmembrane protein 184C
Tmem126a	-1,66	0,000000	transmembrane protein 126A
Dclk3	-1,66	0,000008	doublecortin-like kinase 3
Ap3m1-ps	-1,66	0,000003	adaptor-related protein complex 3, mu 1 subunit, pseudogene [Source:MGI Symbol;Acc:MGI:3645307]

Acly	-1,66	0,000016	ATP citrate lyase
Npc1	-1,66	0,000008	Niemann-Pick type C1; Niemann Pick type C1
Fam102a	-1,67	0,000002	family with sequence similarity 102, member A; family with sequence similarity 102, member A (Fam102a), mRNA.
Ugt2a3	-1,67	0,000000	UDP glucuronosyltransferase 2 family, polypeptide A3
Ces2d-ps	-1,67	0,000022	carboxylesterase 2D, pseudogene
Otc	-1,67	0,000000	ornithine transcarbamylase
Narf	-1,67	0,000000	nuclear prelamin A recognition factor
Mocs1	-1,67	0,000034	molybdenum cofactor synthesis 1
Hhex	-1,67	0,003884	hematopoietically expressed homeobox
Pm20d1	-1,68	0,000000	peptidase M20 domain containing 1
Mmab	-1,68	0,000000	methylmalonic aciduria (cobalamin deficiency) type B homolog (human)
Ces2c	-1,68	0,000047	carboxylesterase 2C
Acpp	-1,68	0,000009	acid phosphatase, prostate
mt-Tr	-1,68	0,000053	mitochondrially encoded tRNA arginine [Source:MGI Symbol;Acc:MGI:102476]
Gpx6	-1,68	0,000000	glutathione peroxidase 6
Cyp2c70	-1,68	0,000050	cytochrome P450, family 2, subfamily c, polypeptide 70
Itih5	-1,69	0,000000	inter-alpha (globulin) inhibitor H5
Tm2d3	-1,69	0,000001	TM2 domain containing 3
Oat	-1,69	0,000121	ornithine aminotransferase
Sult5a1	-1,69	0,000298	sulfotransferase family 5A, member 1
Rpl3-ps1	-1,69	0,000003	ribosomal protein L3, pseudogene 1
Slco1a4	-1,70	0,003942	solute carrier organic anion transporter family, member 1a4; Synthetic construct Mus musculus clone IMAGE:100062527, MGC:190906 solute carrier organic anion transporter family, member 1a4 (Slco1a4) mRNA, encodes complete protein.
Cyp2a4	-1,71	0,001476	cytochrome P450, family 2, subfamily a, polypeptide 4
Aldh8a1	-1,71	0,000002	aldehyde dehydrogenase 8 family, member A1
Fbxw5	-1,72	0,000098	F-box and WD-40 domain protein 5
Mgam	-1,72	0,000000	maltase-glucoamylase
Gls2	-1,72	0,000004	glutaminase 2 (liver, mitochondrial); glutaminase 2 (liver, mitochondrial) (Gls2), nuclear gene encoding mitochondrial protein, mRNA.
Mvd	-1,73	0,000000	mevalonate (diphospho) decarboxylase
Elovl6	-1,74	0,000042	ELOVL family member 6, elongation of long chain fatty acids (yeast)
Usp18	-1,75	0,000030	ubiquitin specific peptidase 18
Lhpp	-1,75	0,000000	phospholysine phosphohistidine inorganic pyrophosphate phosphatase
Rps13-ps2	-1,76	0,000005	ribosomal protein S13, pseudogene 2
Tbc1d30	-1,76	0,000008	TBC1 domain family, member 30
Enpp2	-1,76	0,000014	ectonucleotide pyrophosphatase/phosphodiesterase 2
Antxr2	-1,77	0,000001	anthrax toxin receptor 2
Car5a	-1,77	0,000000	carbonic anhydrase 5a, mitochondrial
Rab9b-ps1	-1,77	0,000087	RAB9B, member RAS oncogene family, pseudogene 1
Ndufb9	-1,77	0,000001	NADH dehydrogenase (ubiquinone) 1 beta subcomplex, 9
Fzd8	-1,80	0,000267	frizzled homolog 8 (Drosophila)
Ugt2b1	-1,83	0,000000	UDP glucuronosyltransferase 2 family, polypeptide B1
Dmgdh	-1,83	0,000000	dimethylglycine dehydrogenase precursor
Lrtm1	-1,83	0,000001	leucine-rich repeats and transmembrane domains 1
Kcnk5	-1,84	0,000059	potassium channel, subfamily K, member 5
Etnppl	-1,85	0,000000	ethanolamine phosphate phospholyase
Dhcr7	-1,86	0,000000	7-dehydrocholesterol reductase
Mr1	-1,87	0,000000	major histocompatibility complex, class I-related
Acss2	-1,87	0,000000	acyl-CoA synthetase short-chain family member 2
Snord13	-1,88	0,006153	small nucleolar RNA, C/D box 13
mt-Tk	-1,89	0,006851	mitochondrially encoded tRNA lysine [Source:MGI

			Symbol;Acc:MGI:102483]
Pcsk9	-1,89	0,000000	proprotein convertase subtilisin/kexin type 9
Scpep1	-1,89	0,000000	serine carboxypeptidase 1
Tstd1	-1,92	0,000000	thiosulfate sulfurtransferase (rhodanese)-like domain containing 1
Cyp2r1	-1,94	0,000000	cytochrome P450, family 2, subfamily r, polypeptide 1; cytochrome P450, family 2, subfamily r, polypeptide 1 (Cyp2r1), mRNA.
Aadat	-1,94	0,000015	aminoadipate aminotransferase
Pdcd4	-1,94	0,000000	programmed cell death 4
Cyp2c68	-1,94	0,000000	cytochrome P450, family 2, subfamily c, polypeptide 68
Cyp2c40	-1,94	0,000000	cytochrome P450, family 2, subfamily c, polypeptide 40
Insig1	-1,96	0,000001	insulin induced gene 1
Slc17a5	-1,97	0,000002	solute carrier family 17 (anion/sugar transporter), member 5
Cyp7b1	-1,98	0,000002	cytochrome P450, family 7, subfamily b, polypeptide 1
Chic1	-1,98	0,000000	cysteine-rich hydrophobic domain 1
Aldoc	-1,98	0,000000	aldolase C, fructose-bisphosphate
Usp2	-2,00	0,000000	ubiquitin specific peptidase 2
Bhmt	-2,00	0,000021	betaine-homocysteine methyltransferase
Cyp2c67	-2,00	0,000001	cytochrome P450, family 2, subfamily c, polypeptide 67
Pmvk	-2,01	0,000000	phosphomevalonate kinase
Nudt7	-2,01	0,003371	nudix (nucleoside diphosphate linked moiety X)-type motif 7
Tlcd2	-2,03	0,000011	TLC domain containing 2
Stard4	-2,04	0,000000	StAR-related lipid transfer (START) domain containing 4
Cyp2c69	-2,04	0,000000	cytochrome P450, family 2, subfamily c, polypeptide 69
Coq5	-2,07	0,000008	coenzyme Q5 homolog, methyltransferase (yeast)
Pstpip2	-2,07	0,000000	proline-serine-threonine phosphatase-interacting protein 2
Foxq1	-2,08	0,010672	forkhead box Q1
Fam213a	-2,08	0,000000	family with sequence similarity 213, member A
Lifr	-2,09	0,000034	leukemia inhibitory factor receptor
Selenbp1	-2,10	0,000015	selenium binding protein 1
Mvk	-2,10	0,000000	mevalonate kinase
Myc	-2,11	0,024083	myelocytomatosis oncogene
Mpv17l	-2,11	0,000000	Mpv17 transgene, kidney disease mutant-like; Mpv17 transgene, kidney disease mutant-like (Mpv17l), nuclear gene encoding mitochondrial protein, mRNA.
Sult1b1	-2,14	0,000000	sulfotransferase family 1B, member 1
Car1	-2,18	0,000000	carbonic anhydrase 1
Slc23a1	-2,19	0,000001	solute carrier family 23 (nucleobase transporters), member 1
Lpin1	-2,20	0,000049	lipin 1
Snord13	-2,21	0,003047	small nucleolar RNA, C/D box 13
Susd4	-2,22	0,000001	sushi domain containing 4
mt-Tf	-2,24	0,010174	mitochondrially encoded tRNA phenylalanine [Source:MGI Symbol;Acc:MGI:102487]
F11	-2,26	0,000000	coagulation factor XI
LOC102631757	-2,26	0,000010	uncharacterized LOC102631757
Urad	-2,27	0,000001	ureidoimidazoline (2-oxo-4-hydroxy-4-carboxy-5) decarboxylase
Gpcpd1	-2,32	0,000336	glycerophosphocholine phosphodiesterase GDE1 homolog (S. cerevisiae) (Gpcpd1), transcript variant 3, mRNA.; glycerophosphocholine phosphodiesterase GDE1 homolog (S. cerevisiae)
Hmgcr	-2,35	0,000000	3-hydroxy-3-methylglutaryl-Coenzyme A reductase
Selenbp2	-2,36	0,000022	selenium binding protein 2
Fdft1	-2,45	0,000000	farnesyl diphosphate farnesyl transferase 1
Acmsd	-2,55	0,000000	amino carboxymuconate semialdehyde decarboxylase
Lss	-2,65	0,000000	lanosterol synthase
Slc46a3	-2,68	0,000000	solute carrier family 46, member 3
mt-Ta	-2,75	0,003469	mitochondrially encoded tRNA alanine [Source:MGI

			Symbol;Acc:MGI:102491]
Cyp17a1	-2,88	0,000001	cytochrome P450, family 17, subfamily a, polypeptide 1
Thrsp	-2,89	0,000002	thyroid hormone responsive
Rdh11	-2,89	0,000000	retinol dehydrogenase 11
Bhmt-ps1	-2,92	0,000001	betaine-homocysteine methyltransferase, pseudogene 1
Papss2	-3,09	0,000000	3-phosphoadenosine 5-phosphosulfate synthase 2; 3'-phosphoadenosine 5'-phosphosulfate synthase 2
Snora69	-3,14	0,000001	small nucleolar RNA, H/ACA box 69
Hsd17b7	-3,16	0,000000	hydroxysteroid (17-beta) dehydrogenase 7
mt-Ts2; mt-Tl2	-3,41	0,034437	mitochondrially encoded tRNA serine 2 [Source:MGI Symbol;Acc:MGI:102474]; mitochondrially encoded tRNA leucine 2 [Source:MGI Symbol;Acc:MGI:102481]
Hmgcs1	-3,74	0,000000	3-hydroxy-3-methylglutaryl-Coenzyme A synthase 1
Cyp51	-4,00	0,000000	cytochrome P450, family 51
Nsdhl	-4,47	0,000000	NAD(P) dependent steroid dehydrogenase-like
Fdps	-4,50	0,000000	farnesyl diphosphate synthetase
Clec2h	-4,61	0,000002	C-type lectin domain family 2, member h
Msmo1	-4,69	0,000000	methylsterol monoxygenase 1
Adgrf1	-6,39	0,000000	adhesion G protein-coupled receptor F1
Idi1	-7,28	0,000000	isopentenyl-diphosphate delta isomerase
Moxd1	-10,08	0,000338	monooxygenase, DBH-like 1
Sqle	-10,26	0,000000	squalene epoxidase

Supplementary Table 2. Differentially expressed genes in liver tissue of TAT-Gap19-treated mice. RNA was extracted from liver tissue of TAT-Gap19-treated and saline-treated mice, and whole transcriptome microarray analysis was performed using Affymetrix Clariom™ D mouse arrays. Background correction, summarization and normalization of all data were done with Expression Console and Affymetrix Transcriptome Analysis Console Software. Fold change threshold was set at <-1.5 and >1.5, and results were statistically processed by 1-way analysis of variance.

Gene symbol	Fold change	p-value	Description
Snora41	2,27	0,000016	small nucleolar RNA, H/ACA box 41
Snora36b; Mir664	2,03	0,015950	small nucleolar RNA, H/ACA box 36B; microRNA 664
Tff3	2,01	0,001641	trefoil factor 3, intestinal
Mir669b	1,99	0,028966	microRNA 669b
Scarna6	1,99	0,001413	small Cajal body-specific RNA 6
Rnu2-10	1,74	0,003643	U2 small nuclear RNA 10
Thrsp	1,74	0,011785	thyroid hormone responsive
Rnu2-10	1,73	0,004916	U2 small nuclear RNA 10
Rnu12	1,72	0,045477	RNA U12, small nuclear
Mir505	1,69	0,000064	microRNA 505
Snord71	1,67	0,000033	small nucleolar RNA, C/D box 71
Snora7a	1,65	0,001927	small nucleolar RNA, H/ACA box 7A
Selenbp2	1,63	0,013367	selenium binding protein 2
Snord49a	1,60	0,000157	small nucleolar RNA, C/D box 49A
Snord14e	1,57	0,004053	small nucleolar RNA, C/D box 14E
Cyp7b1	1,57	0,009359	cytochrome P450, family 7, subfamily b, polypeptide 1
Obox4-ps3	1,54	0,021734	oocyte specific homeobox 4, pseudogene 3 [Source:MGI Symbol;Acc:MGI:5521023]
Rb1	1,54	0,017943	retinoblastoma 1
Mir15a	1,53	0,002577	microRNA 15a
Ctsc	-1,53	0,008574	cathepsin C
Cd53	-1,53	0,000183	CD53 antigen
Folr2	-1,53	0,006662	folate receptor 2 (fetal)
Hmox1	-1,53	0,000056	heme oxygenase 1; heme oxygenase (decycling) 1
Acot3	-1,54	0,009736	acyl-CoA thioesterase 3
Cybb	-1,56	0,000188	cytochrome b-245, beta polypeptide
Adgre1	-1,57	0,001940	adhesion G protein-coupled receptor E1
Gsta2	-1,58	0,003164	glutathione S-transferase, alpha 2 (Yc2)
Ifi27l2b	-1,60	0,015937	interferon, alpha-inducible protein 27 like 2B
Gpnmb	-1,61	0,000055	glycoprotein (transmembrane) nmb

Gtpbp4-ps4	-1,62	0,003893	GTP binding protein 4, pseudogene 4 [Source:MGI Symbol;Acc:MGI:5521103]
Lilr4b	-1,62	0,000120	leukocyte immunoglobulin-like receptor, subfamily B, member 4B
Ly86	-1,62	0,000075	lymphocyte antigen 86
Vsig4	-1,64	0,023868	V-set and immunoglobulin domain containing 4
Lyz1	-1,66	0,000126	lysozyme 1
Cd68	-1,67	0,000017	CD68 antigen (Cd68), mRNA.; CD68 antigen
Cd5l	-1,69	0,006313	CD5 antigen-like
Ctss	-1,69	0,000229	cathepsin S; cathepsin S (Ctss), transcript variant 1, mRNA.
Fcer1g	-1,75	0,000045	Fc receptor, IgE, high affinity I, gamma polypeptide
Lilrb4a	-1,76	0,000019	leukocyte immunoglobulin-like receptor, subfamily B, member 4A
Wfdc17	-1,80	0,000416	WAP four-disulfide core domain 17
Mmp12	-1,82	0,000009	matrix metallopeptidase 12
C1qb	-1,87	0,000174	complement component 1, q subcomponent, beta polypeptide
Serpina7	-1,91	0,022512	serine (or cysteine) peptidase inhibitor, clade A (alpha-1 antiproteinase, antitrypsin), member 7
Cd36	-1,92	0,006360	CD36 antigen
Igfbp1	-1,94	0,046974	insulin-like growth factor binding protein 1
Clec7a	-2,06	0,000050	C-type lectin domain family 7, member a
Lyz2	-2,17	0,000044	lysozyme 2
Mfsd2a	-2,26	0,004871	major facilitator superfamily domain containing 2A
Gtpbp4-ps1	-2,29	0,001300	GTP binding protein 4, pseudogene 1 [Source:MGI Symbol;Acc:MGI:5521100]

Supplementary Table 3. Differentially expressed genes in liver tissue of TAT-Gap24-treated mice. RNA was extracted from liver tissue of TAT-Gap24- and saline-treated mice and whole transcriptome microarray analysis was performed using Affymetrix Clariom™ D mouse arrays. Background correction, summarization and normalization of all data were done with Expression Console and Affymetrix Transcriptome Analysis Console Software. Fold change threshold was set at <-1.5 and >1.5, and results were statistically processed by 1-way analysis of variance.

Gene symbol	Fold change	p-value	Description
Snora62	1,83	0,016622	small nucleolar RNA, H/ACA box 62
Mir5125	1,76	0,011733	microRNA 5125
mt-Tk	1,76	0,049245	mitochondrially encoded tRNA lysine [Source:MGI Symbol;Acc:MGI:102483]
Rnu12	1,72	0,026838	RNA U12, small nuclear
Egln3	1,70	0,023996	EGL nine homolog 3 (C. elegans), mRNA (cDNA clone MGC:36685 IMAGE:5371854), complete cds.
Scarna6	1,59	0,012201	small Cajal body-specific RNA 6
Mir466f-4	1,57	0,003358	microRNA 466f-4 [Source:MGI Symbol;Acc:MGI:3783374]
Rb1	1,53	0,002388	retinoblastoma 1
Mir15a	1,53	0,009847	microRNA 15a
Mlxipl	1,53	0,012992	MLX interacting protein-like; Synthetic construct Mus musculus clone IMAGE:100068140, MGC:195757 MLX interacting protein-like (Mlxipl) mRNA, encodes complete protein.
Mir5125	1,51	0,015165	microRNA 5125
Snord4a	-1,51	0,000624	small nucleolar RNA, C/D box 4A
Lyz2	-1,59	0,000379	lysozyme 2
Snord11	-1,63	0,000496	small nucleolar RNA, C/D box 11
Clec7a	-1,66	0,017295	C-type lectin domain family 7, member a
Snora34; Mir1291	-1,72	0,001410	small nucleolar RNA, H/ACA box 34; microRNA 1291
Wfdc17	-1,84	0,000221	WAP four-disulfide core domain 17

Supplementary Table 4. Primers used for qRT-PCR analysis.

Gene	Assay ID	Accession number	Assay location	Amplicon size (base pairs)	Exon boundary
Gja1	Mm01179639_s1	NM_010288.3	2937	168	2-2
Gjb2	Mm00433643_s1	NM_008125.3	603	72	2-2
Gjb1	Mm01950058_s1	NM_008124.2	466	65	1-1
18S	Hs99999901_s1	X03205.1	604	187	1–1
Actb	Mm00607939_s1	NM_007393.3	1233	115	6–6
B2m	Mm00437762_m1	NM_009735.3	111	77	1–2
Gapdh	Mm99999915_g1	NM_008084.2	265	107	2–3
Hmbs	Mm01143545_m1	NM_013551.2	473	81	6–7
Ubc	Mm02525934_g1	NM_019639.4	370	176	2–2

Supplementary Figure 1. Effects of TAT-Gap24 and TAT-Gap19 on hepatocyte viability. MTT test after exposure of TAT-Gap24 and TAT-Gap19 for 24 h and 48 h. Results were normalized to 100%, which corresponds with values at 0 µM, and data are expressed as means ± SEM.

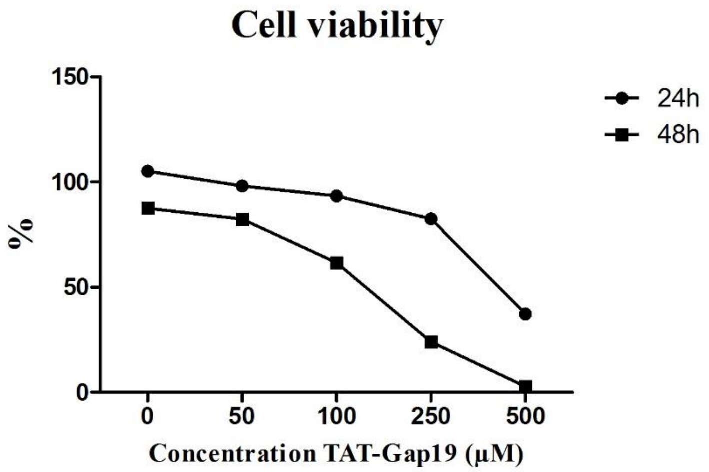

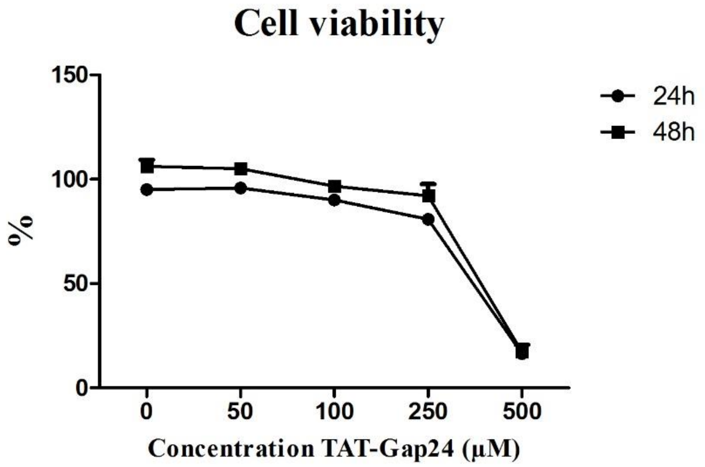

Supplementary Figure 2. Effects of CHFD on connexin mRNA and protein expression in liver. Immunoblot analysis of Cx26 (21 kDa), Cx32 (27 kDa) and Cx43 (43 kDa) after separation and blotting after which results were normalized to total protein. Blot images are cropped. Full-length blots are presented in Supplementary Figure 4. Data are expressed as means ± SEM with *p<0.5 and **p<0.01.

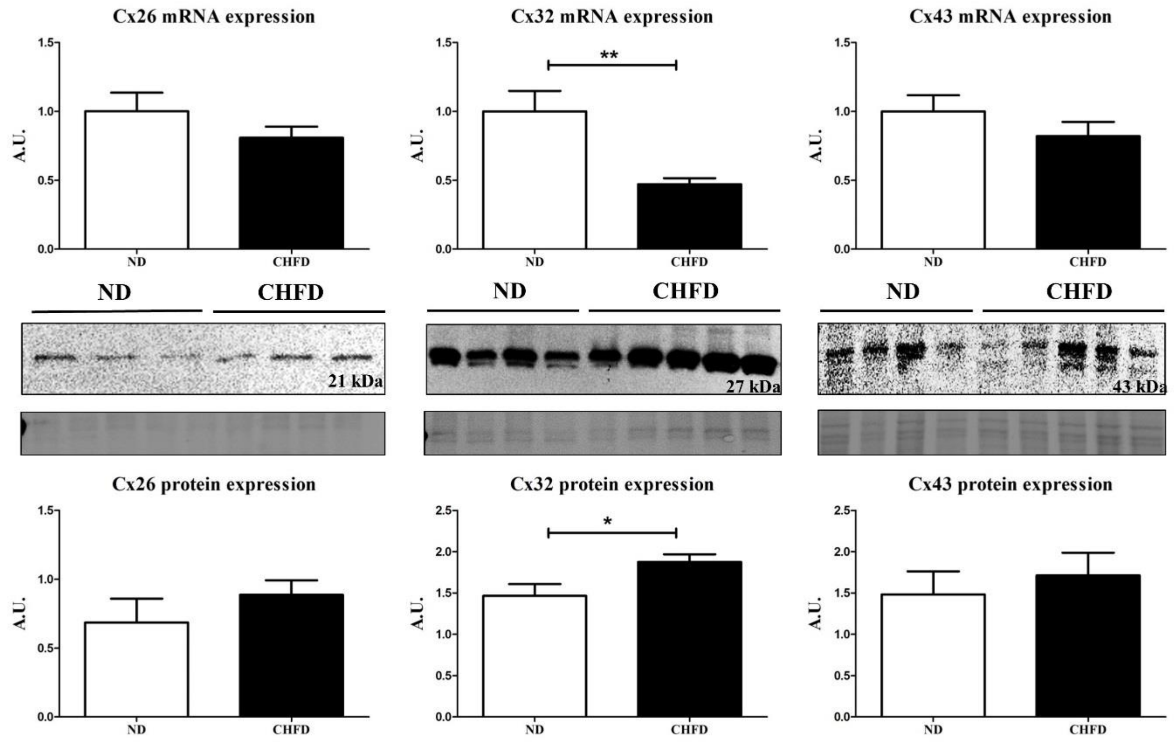

Supplementary Figure 3. Full-length blots of Supplementary Figure 2. Effects of CHFD on connexin mRNA and protein expression in liver. Immunoblot analysis of Cx26 (21 kDa), Cx32 (27 kDa) and Cx43 (43 kDa) after separation and blotting, after which results were normalized to total protein.

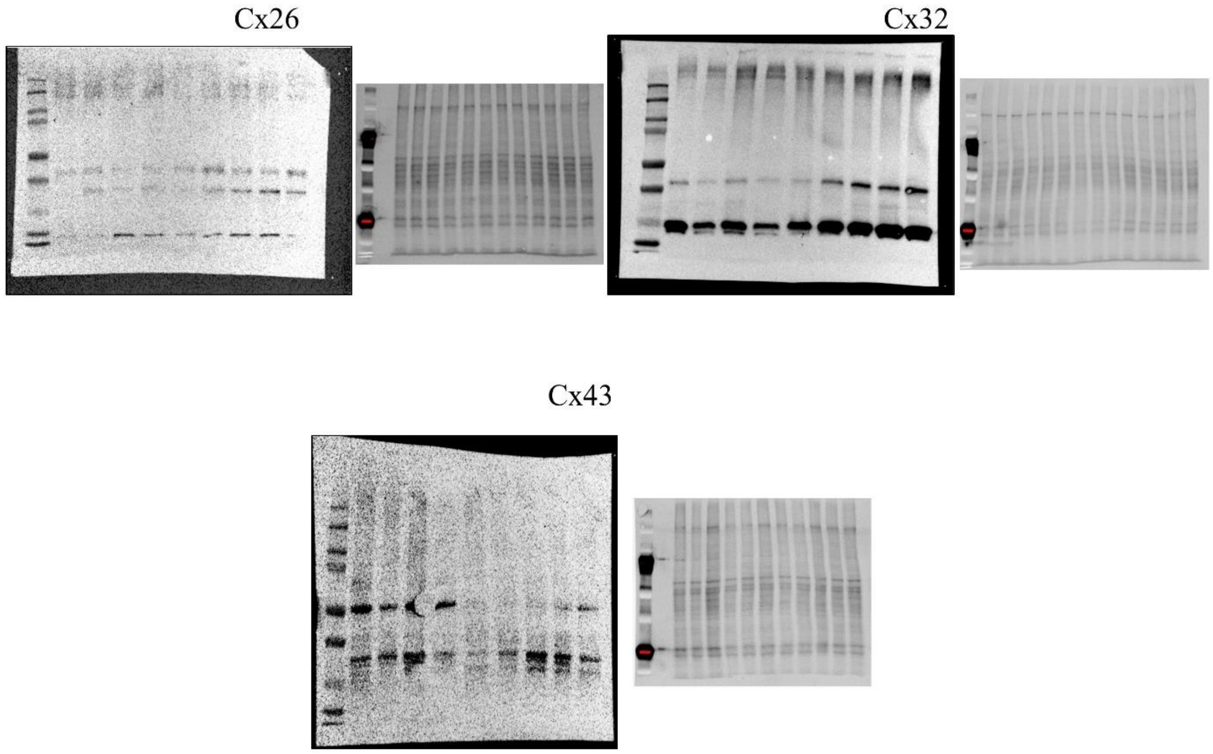

Supplementary Figure 4. Full-length blots of Figure 2. Effects of TAT-Gap24 and TAT-Gap19 on connexin protein expression in liver. Immunoblot analysis of Cx26 (21 kDa), Cx32 (27 kDa) and Cx43 (43 kDa) after separation and blotting, after which results were normalized to total protein loading.

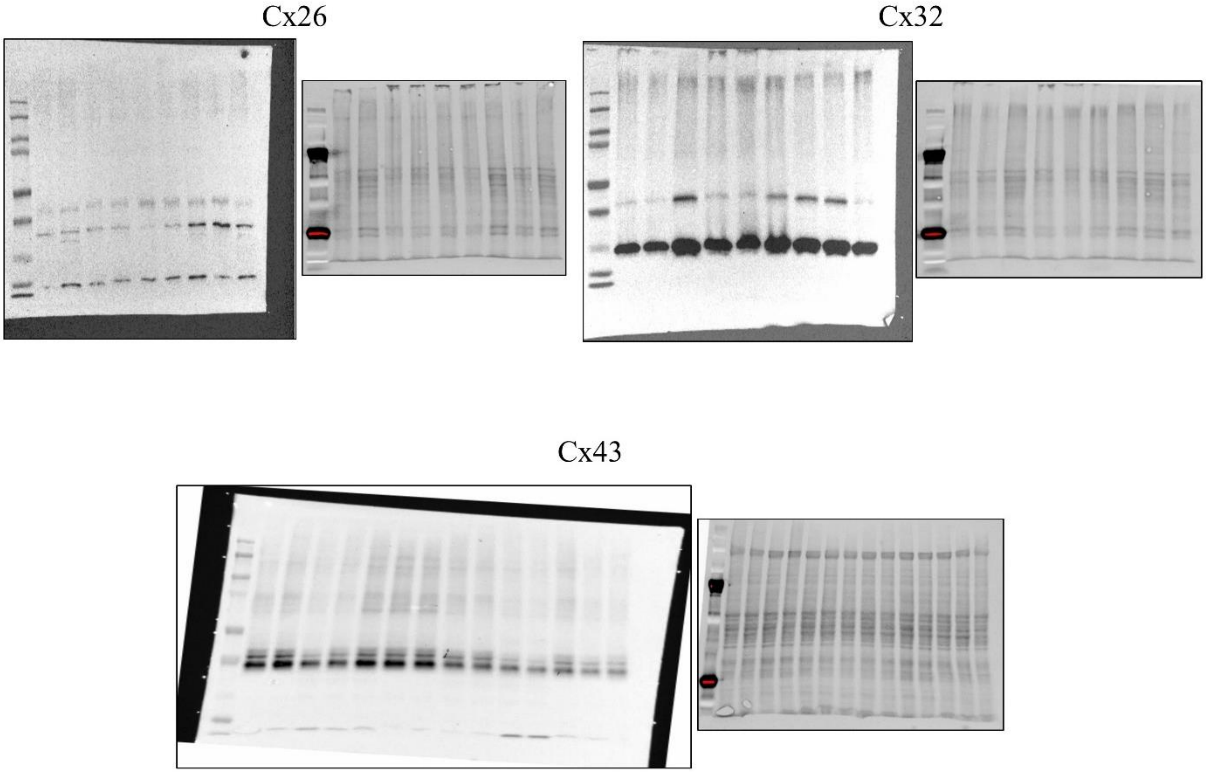

Supplementary Figure 5. Full-length blots of Figure 6. Protein expression analysis of liver tissue of TAT-Gap24 and TAT-Gap19-treated NASH animals. Immunoblot analysis of CD36 (53 kDa), NADPH oxidase (65 kDa) and LY86 (24 kDa) after separation and blotting, after which results were normalized to total protein.

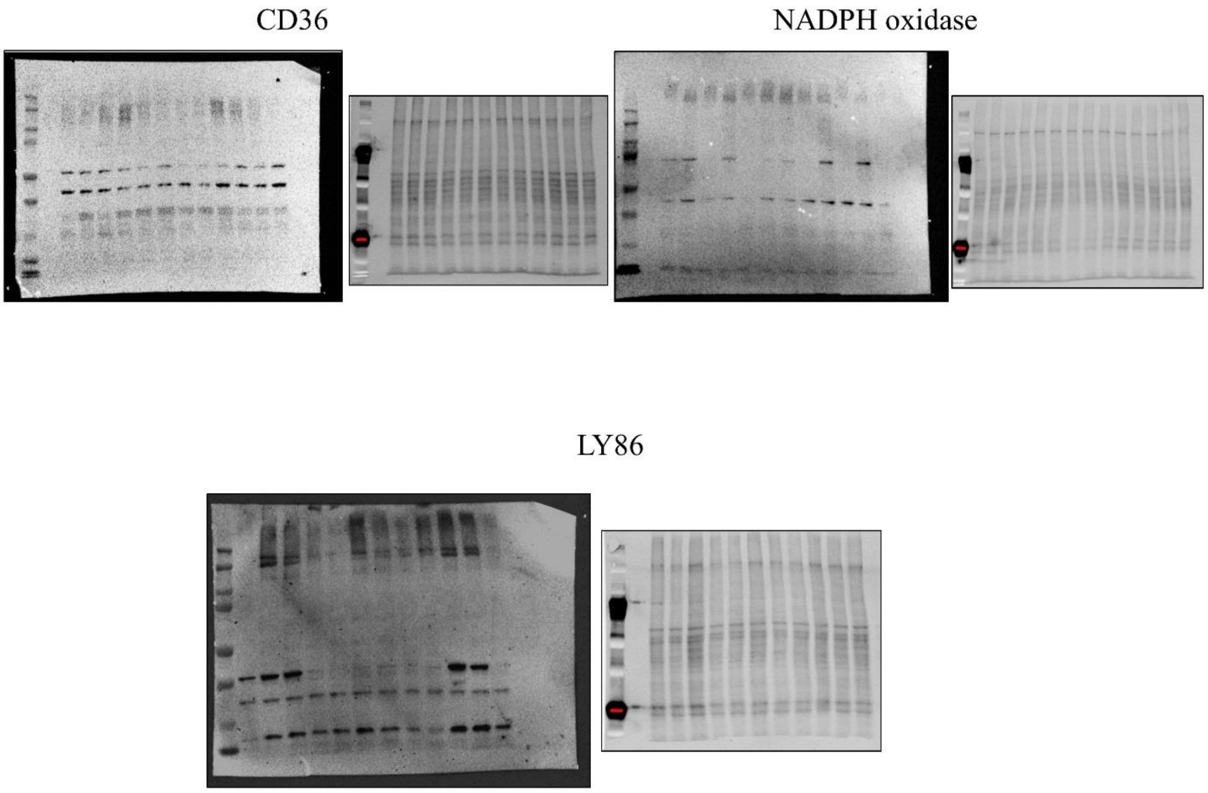

Supplement: Supplementary file 1 — Supplementary data [file 41598_2017_8583_MOESM1_ESM.pdf]
